# Strategies and Mechanisms of Thermal Compensation in Newborn Water Buffaloes

**DOI:** 10.3390/ani13132161

**Published:** 2023-06-30

**Authors:** Daniel Mota-Rojas, Ada Braghieri, Marcelo Ghezzi, María Carolina Ceriani, Julio Martínez-Burnes, Pamela Anahí Lendez, Alfredo M. F. Pereira, Karina Lezama-García, Adriana Domínguez-Oliva, Alejandro Casas-Alvarado, Emilio Sabia, Corrado Pacelli, Fabio Napolitano

**Affiliations:** 1 Neurophysiology, Behavior and Animal Welfare Assessment, DPAA, Universidad Autónoma Metropolitana (UAM), Mexico City 04960, Mexico; 2 Scuola di Scienze Agrarie, Forestali, Alimentari ed Ambientali, Università degli Studi della Basilicata, 85100 Potenza, Italy; 3 Animal Welfare Area, Faculty of Veterinary Sciences (FCV), Universidad Nacional del Centro de la Provincia de Buenos Aires (UNCPBA), University Campus, Tandil 7000, Argentina; 4 Faculty of Veterinary Sciences, Universidad Nacional del Centro de la Provincia de Buenos Aires (UNCPBA), Tandil, Veterinary Research Center (CIVETAN), CONICET-CICPBA, Arroyo Seco S/N, Campus Universitario, Tandil 7000, Argentina; 5 Facultad de Medicina Veterinaria y Zootecnia, Universidad Autónoma de Tamaulipas, Victoria City 87000, Mexico; 6 Mediterranean Institute for Agriculture, Environment and Development (MED), Universidade de Évora, Pólo da Mitra, Ap. 94, 7006-554 Évora, Portugal; 7 School of Agricultural, Forest, Food, and Environmental Sciences, University of Basilicata, 85100 Potenza, Italy

**Keywords:** *Bubalus bubalis*, ruminant hypothermia, thermoregulation, shivering thermogenesis, non-shivering thermogenesis

## Abstract

**Simple Summary:**

Although ruminant newborns are a precocial species, they are susceptible to hypothermia and neonatal mortality during the first hours of life due to intrinsic and extrinsic factors that challenge their thermostability. Several mechanisms are activated when the organism perceives a temperature decrease, such as vasoconstriction, shivering, and non-shivering thermogenesis. These have been widely studied in lambs, goats, and cattle. However, for water buffalo, a relevant, productive species, the investigation of thermoregulation in the neonate immediately after calving, and of the tools to assess their thermal state, is limited. This review aims to analyze behavioral, morphological, and physiological strategies in newborn water buffaloes facing thermal stressors and discuss the role of infrared thermography in monitoring hypothermic states.

**Abstract:**

Hypothermia is one of the principal causes of perinatal mortality in water buffaloes and can range from 3% to 17.9%. In ruminants, factors affecting hypothermia in newborns may be of intrinsic (e.g., level of neurodevelopment, birth weight, vitality score, amount of brown fat, skin features) or extrinsic origin (e.g., maternal care, environmental conditions, colostrum consumption). When newborn buffaloes are exposed to cold stress, thermoregulatory mechanisms such as peripheral vasoconstriction and shivering and non-shivering thermogenesis are activated to prevent hypothermia. Due to the properties of infrared thermography (IRT), as a technique that detects vasomotor changes triggered by a reduction in body temperature, evaluating the central and peripheral regions in newborn buffaloes is possible. This review aims to analyze behavioral, physiological, and morphological strategies and colostrum consumption as thermal compensation mechanisms in newborn water buffalo to cope with environmental changes affecting thermoneutrality. In addition, the importance of monitoring by IRT to identify hypothermia states will be highlighted. Going deeper into these topics related to the water buffalo is essential because, in recent years, this species has become more popular and is being bred in more geographic areas.

## 1. Introduction

Hypothermia in newborn animals is one of the most relevant problems during the perinatal period. It causes severe physiological changes in the animal induced by thermoregulation mechanisms to restore thermoneutrality. Hypothermia in newborn ruminants is considered one of the main problems that increase neonatal mortality in livestock [1,2,3]. 

In the water buffalo (*Bubalus bubalis*), mortality rates in newborns range from 3 to 17.9% [4,5,6]. Hypothermia occurs mainly for intrinsic and extrinsic causes [7], such as deficient or null colostrum intake, low birth weight, exposure to low temperature, and amniotic fluid on the skin. [3,8,9,10].

The multifactorial origin of hypothermia in the newborn challenges the thermoregulation of the neonatal animal and success in reaching its thermoneutrality would depend on the effectiveness of different animal mechanisms [2,11]. However, it is necessary to state that the water buffalo has anatomic–physiological characteristics that differ from domestic bovines, such as the thickness of its skin and the melanin content in the dermis, which possibly makes the ability to lose heat deficient in the water buffalo [12,13,14], giving a sort of resilience to hypothermia. Even though the evidence is clear on these skin characteristics, recognizing this state for early care cannot be ignored.

Infrared thermography (IRT) has been suggested as a method for recognizing hypothermia in domestic animals because it allows evaluation of the surface temperature, which correlates with body temperature [15,16]. However, there is controversy about its use due to the sensitivity of the different central and peripheral thermal windows that can decrease or improve the effectiveness of this tool [11,12,13,17,18,19]. For this reason, the objective of this review is to analyze behavioral, physiological, and morphological strategies, and the importance of colostrum consumption as a thermal compensation mechanism, in newborn water buffaloes to cope with environmental changes that affect their thermoneutral state, as well as to discuss the importance of IRT monitoring to identify hypothermic states [3,4,11].

## 2. Hypothermia in Newborn Ruminants

Mortality in newborn ruminants is a global problem that affects production units and causes biological and economic losses, with a direct impact on the profitability of farms [20]. Perinatal mortality is between 2.4 and 9.7% in dairy cattle [20], between 15 and 20% in lambs [21,22], and between 3 and 17.9% in water buffaloes [4,6]. The most common causes of perinatal mortality include dystocic deliveries, infections, and hypothermia [20]. However, mortality due to hypothermia constitutes only 5% in ruminants [23]. Khounsy et al. [24] pointed out that in large ruminants, it can cause losses of up to USD 2 million a year, with an impact not only on the health of the animal but also on the productivity of the farms. In addition, this phenomenon is one of the main challenges that the newborn faces when beginning its extrauterine life, especially if one takes into account that in the case of the water buffalo it is suggested that the newborn’s body temperature at birth can decline between 10 and 20 °C [25,26,27]. The average rectal temperature in water buffaloes is 38.5 °C to 38.6 °C [28]. The fetal temperature depends upon the mother until birth. Therefore, maternal central temperature alterations can affect the thermal state of the fetus. Maternal heat is transferred through the placenta, resulting in a fetal temperature above the maternal by 0.3 to 0.5 °C. At birth, the newborn loses the maternal regulation and can rapidly reduce its temperature [28,29]. In lambs, a decrease to 1.5 °C lower than that of the intrauterine environment is described [30,31], while in piglets this decrease can reach 2 °C [18,32]. So, it is clear that this phenomenon has a severe productive and physiological impact on the newborn ruminant. However, this can be prevented with a proper understanding of the factors that cause it, which would help in taking timely therapeutic actions [10].

The physiological effects of hypothermia are mainly related to the activation of the autonomic nervous system (ANS) through the activation of catabolic systems to optimize energy resources and thus increase heat production [25]. For example, in an experimental rodent model, it was suggested that exposure to an environmental temperature of 4 °C can lead to the activation of sympathetic adrenergic neurons. This allows the neurosecretion of epinephrine and norepinephrine [11,17]. This effect can be considered the leading cause of the rapid mobilization of glucose reserves, not only to raise the temperature but also to be able to stand up [1]. Similarly, Olson et al. [33] evaluated 17 Holstein-Friesian calves which were exposed to a cold test by immersing them in water at a temperature between 15 and 17 °C, and they observed that body temperature decreased by 10 °C, and glucose increased in parallel with the increase in catecholamines. These results show that activating the sympathetic nervous system (SNS) allows for coordinating the compensatory response to hypothermia. This is complemented by what was observed in a comparative study of the response to hypothermia in water buffalo calves and domestic bovines. Water buffalo calves had at least 20 mg more triglycerides than domestic bovine calves after exposure to environmental temperatures of 15 °C, which indicates an increased expenditure of energy resources due to SNS activation. However, water buffalo calves were also found to have 2 °F (16 °C) lower rectal temperatures [34]. These authors’ results possibly show that the newborn’s body temperature drop was caused by optimizing their resources to increase heat production. However, it is necessary to point out that this response does not translate into the animal being able to successfully compensate for the low temperature, due to the limited availability of resources, which can lead to opting for alternative metabolic routes, such as anaerobic glycolysis that generates less efficient products and can lead to acidosis [25]. These facts confirm the importance of recognizing and treating hypothermia in the newborn.

On the other hand, the activation of the SNS causes changes in the increased mobilization of energy resources. Mechanisms are also activated at the vascular level in response to the release of catecholamines that cause a reduction in the caliber of peripheral blood vessels, to avoid heat loss to the environment [11]. Although this mechanism may be a favorable change in a thermoregulatory sense, it could also bring consequences due to the limited oxygen supply in the brain [35]. This was described by Tyler and Ramsey [36], who evaluated the effect of reducing blood oxygen in 12 Holstein calves during 72 h postpartum. The authors found that, due to hypoxia, CO_2_ levels did not exceed 45 mmHg and glucose concentrations tended to be lower in hypoxic calves (110 mg/dL vs. 105 mg/dL, *p* > 0.1). However, lactate was higher between 6 and 42 h postpartum in animals with hypoxia, and concentrations between 90 and 23 mg/dL were observed during that period (*p* = 0.01). These results show that during hypothermia, a state of hypoxia can be generated in the neonate and, consequently, it will require an increase in the rate of heat production. In addition, in the absence of oxygen, it will be necessary to resort to anaerobic metabolic pathways, giving rise to high lactate levels and a tendency to respiratory and metabolic acidosis, which is understood as a valuable biomarker of health status in the neonate. However, the drop in oxygen might also promote a reversal of the partial pressure of carbon dioxide, leading to respiratory alkalosis [1,25,35,36,37,38,39,40,41,42,43].

**Figure 1:**
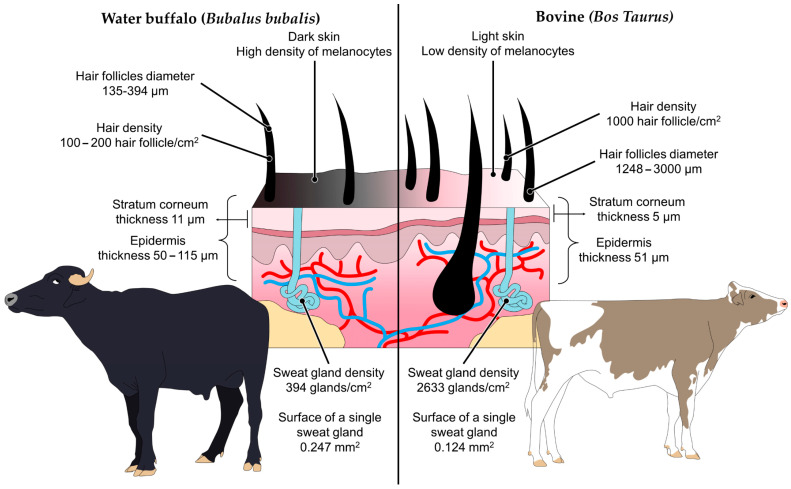
Differences in dermal structure between *Bubalus bubalis* and *Bos taurus*. Water buffaloes have a thicker epidermis and stratum corneum, as well as more melanocytes, giving them dark skin. These are properties that contribute to providing resistance to cold in adult animals. However, the density and size of sweat glands, as well as the number of hair follicles, makes them susceptible to heat stress in adulthood.

In summary, hypothermia in a newborn water buffalo generates positive catabolic changes that help increase heat production due to the increased tone of the SNS. This can cause vasomotor changes that would limit oxygen supply to the brain, promoting necrosis in this tissue. This is due to a decrease in the local circulation due to hypertension. This can also cause changes in local metabolism by triggering anaerobic glycolysis, resulting in cerebral tissue necrosis, a deadly outcome for newborns. Moreover, hypothermia can be multifactorial. For example, a study by Hancock et al. [7] evaluated eight herds with 440 lambs of the Ideal (Polwarth), Corriedale, and Romney Marsh breeds, and showed that the origin of hypothermia does not come from a single etiology but rather is triggered by various factors that come together to challenge the effectiveness of the different thermoregulation mechanisms (Figure 1) [39,40,41]. The associated factors include the size of the litter (twins and triplets), poor maternal care, and no colostrum intake [10,20,23,44,45].

An interesting factor to consider is offspring care, which may include licking the newborn, helping to remove fetal membranes, but Whalin et al. [46] mention that licking can also stimulate mechanoreceptors, which would help to stimulate the respiration (reducing the chance of initiating hypoxia) and vigor of the calf, which is possibly related to the increase in its temperature. Barrier et al. [47] mentioned that a calf´s vigor depends on the mother´s care at birth in Holstein cows and observed that the calves receiving assistance from an operator took longer to stand up, walk, and suckle from the mother than the animals that did not have assistance during calving. In contrast, their mother spent more time licking those newborns. Although this makes it clear that poor maternal care is a possible contributing factor in hypothermia, it may have a greater impact on direct mortality. In addition, it is important to mention that the water buffalo is a precocious species, so its thermoregulation mechanisms may differ in anatomical–physiological characteristics that can help reduce heat loss or gain [25]. Specifically, water buffalo are described as having unique skin characteristics that give the animal a resilience to hypothermia.

Therefore, the scientific evidence demonstrates that hypothermia generates negative metabolic, physiological, and hemodynamic changes in the body of the newborn, which reaffirms its potential impact on the health of the newborn water buffalo. However, it is necessary to explore the different factors that promote hypothermia in newborn buffaloes in order to understand the compensation mechanisms for this state and the possible physiological repercussions when newborns fail to return to thermoneutrality [48,49,50,51,52].

## 3. Intrinsic Factors Affecting the Temperature in the Newborn Buffalo

Nasr [53] recorded a 35% incidence of stillbirths in primiparous Egyptian buffaloes, compared to crosses of Egyptian buffalo with Italian buffalo and Egyptian buffalo crosses, in which percentages of 10.60% and 4.5% were observed, respectively. Likewise, long-lasting gestations longer than 321 days were observed in pure Egyptian breeds compared to Italian buffalo, where the duration decreased. However, in canids, this effect of a prolonged gestational time could result in a higher weight of the neonates and dystocia at parturition, generating a drop in survival rates, where one of the main associated factors is low temperatures.

On the other hand, within the factors attributable to breeding, we can mention low birth weight, which implies alterations in blood biomarkers, which have been correlated in the same way with the mother´s weight in canine puppies [54,55]. In cattle, the lack of mother–calf bond (imprinting) causes 50% of deaths due to hypothermia. Dubey et al. [39] observed that mothers spent more time *(p* < 0.05) sniffing and licking the calf´s body in the first half hour after parturition, which favors the recognition of the calf and the establishment of a strong bond between them. They observed that licking the calf allows the mother not only to dry her offspring´s fur and avoid evaporative cooling but also to promote the activity of the calf and stimulate the respiratory center, circulation, urination, and defecation. Moreover, it can help the calf get up in a period of 45 min and connect with the teat for approximately 4 h [39]. These abilities together avoid thermal shock due to external weather [40,41].

Lezama García et al. [56] and Olson et al. [33] related the efficient thermoregulation of the newborn to individual characteristics, such as body reserves of energy components at birth, made up of labile proteins, lipids, and liver and muscle glycogen. Likewise, other factors can be mentioned, such as the presence of amniotic fluid on the skin, since this fluid can lead to heat loss due to its evaporation, and it covering large areas of the body surface can significantly reduce the temperature of the individual. To all of the above we add the aspiration of fluid in the respiratory tract that limits the animal’s respiration [1,57,58], which could make the mechanisms of temperature compensation less efficient due to the limitation of critical energetic substrates such as oxygen [43]. However, to clearly understand the influence of these factors, they must be discussed individually.

### 3.1. Birth Weight

Birth weight can be considered one of the most important factors contributing to the development of hypothermia. In fact, in domestic animals, it has been possible to show the impact of low birth weight on thermoregulatory capacity in neonatal dog puppies [55,56], pigs [32], sheep [7,8], bovines [1,35], and water buffalo [3]. This effect may impact the vitality of the newborn calf [59], and due to low vitality, their ability to consume colostrum may be limited, both in the amount of colostrum ingested and the time lag between parturition and first suckling [60,61]. In addition, birth weight may have an influence on the amount and proportion of brown adipose tissue (BAT) in the weight of neonates, and a low weight can cause the amount of BAT to be less than 20 g/kg of body weight in newborn cattle and 14 g/kg in the case of sheep [62,63,64].

In pigs, Vazquéz-Mandujano et al. [65] evaluated the effect of birth weight on energy balance in 10 piglets. These authors observed that piglets with low birth weights digested more nitrogen and dry matter compared to animals with higher weights. Likewise, it was observed that energy digestibility was 1.3% more elevated in higher-weight piglets than in low-weight animals, and metabolizable energy was 1% more in these same piglets. In this way, the results shown by these authors would help to support the idea that an animal with a low birth weight would have less energy capacity due to the limited availability of resources to maintain its thermogenesis. Interestingly, this idea is complemented by the study carried out by Barreto et al. [66] where they determined the vitality of 54 Suffolk lambs using the APGAR (appearance, pulse, grimace, activity, and respiration) method. Although these authors observed that low environmental temperature (minimum of 8.75 °C) had a greater impact on the body temperature of the offspring, they also observed that animals weighing less than 3 kg (from multiple births) had a greater chance of developing hypothermia. According to these authors, 62.5% of the cases of deaths due to hypothermia may be due to a state of severe hypoglycemia, and it was even observed that birth weight presented a positive correlation with the APGAR score (r = 0.39, *p* = 0.0015). Thus, it is possible to suggest that animals with a lower weight may not only be associated with a lower thermoregulatory capacity, but also an animal with less vigor, making it difficult to perform other activities, such as standing up. Likewise, a study carried out on 115 Scottish Blackface and Suffolk lambs, evaluating the effect of birth weight and litter size on thermal maintenance capacity, found that lambs with the lowest birth weight had a rectal temperature lower than that of heavier lambs. In addition, heavier animals suckled colostrum for longer time periods than lower-weight animals [67]. The scientific evidence is clear that an animal with low weight may take longer to stand up and therefore be unable to consume colostrum shortly after birth.

The relationship between thermoregulatory capacity and birth weight has been confirmed by Vicente-Pérez et al. [68], using infrared thermography (IRT). They observed a positive correlation between the surface temperature and the weight of the calf at birth (r = 0.25–0.27). In the case of the water buffalo, a similar result is reported, in which the surface temperatures of the thermal windows of the eye, lacrimal caruncle, and auricular pavilion were at least 1 °C lower in animals weighing less than 40 kg [3]. This reaffirmed that the weight is directly related to the newborn animal´s thermoregulatory capacity. In dogs, it has even been suggested that low birth weight can increase the probability of perinatal death by 2.6% and the necessity of post-natal assistance by 2.7 times [69]. For this reason, early recognition of hypothermia is necessary to provide the necessary assistance, as is the case with the consumption of colostrum, which can be a valuable energy resource for newborns [66,68]. Consumption of colostrum in newborns will provide enough energetic substances to complement the available reserves. For example, in lambs it is suggested that colostrum can provide 2 Kcal of energy per mL [70]. Even when it has limited energy resources due to a low birth weight, a newborn can resort to the consumption of colostrum, which would lead to an increase in its energy reserves to recover its thermoneutrality.

In summary, birth weight is directly related to the thermoregulation capacity in the newborn since this would be an indirect measure of the availability of energy resources. In addition, it would limit the ability to perform other actions such as standing up and, therefore, the newborn would require more time to initiate colostrum consumption.

### 3.2. Skin Features

The skin plays an important role in the thermoregulation of animals because this structure can help detect changes in environmental temperature. Skin presents transient receptor potential (TRP) thermoreceptors [71], within which is the TRPM8 that is activated when the environmental temperature is less than 15 °C [72]. Another way the skin acts as the pathway for heat loss to the environment is through convection and heat evaporation [73]. These mechanisms may differ depending on the presence of hair, the structure of the skin, the number of sweat glands, the number of follicles, and the amount of melanin, in ways that can make thermoregulation capacity less efficient [13,74]. Bazzaz [75], comparing the skin of the neck regions of cattle, sheep, and water buffalo, observed that large ruminants (bovine and water buffalo) have fewer sensory nerve fibers than those observed in sheep. Therefore, it could be that the cold threshold in large ruminants is higher compared to other species.

The water buffalo presents a different skin thickness compared with the domestic bovine. This could be because the distribution of the layers that constitute the epidermis, dermis, and hypodermis, is modified depending on the species, habitat, body region, and even stage of life. In the case of adult animals, the presence of a thick epidermis is mentioned, with a prominent stratum corneum of 11 µm, which is different from bovines where it measures 5 µm (Figure 1) [76]. In this regard, Ibrahim and Hussin [77] performed a histological evaluation of the thickness of the stratum corneum in water buffaloes and found large amounts of vascularized connective tissue and numerous bands of collagen fibers in the skin of these animals. With this evidence, it is clear that the buffalo´s skin is at least 50% thicker compared to the domestic bovine. There are regions with thin skin and abundant blood capillaries that could function as thermal windows because they facilitate heat loss or gain [78,79]. However, these thermal windows are insufficient for constant heat loss to be observed, as seen in piglets or sheep [2,32]. In addition, the thickness of the skin in the water buffalo may depend upon the age of the animal. Muralidharan and Ramesh [80], evaluating the dermal characteristics of the skin in newborn buffaloes up to 6 months of age from the Murrah and Gradede Murrah breeds, found a collagen content of 50 ± 0.44 and 44.6 ± 0.34 mg/cm^2^ of skin, respectively. So, from a comparative perspective the newborn water buffalo’s skin can be thinner than the adult´s. However, a relevant issue is the number of hair follicles present during the neonatal stage, as pointed out by Hafez et al. [76], who carried out a morpho-histological typing of the skin in water buffaloes and Egyptian bovines and observed that the water buffalo has an average of 394 follicles/cm2 during the adult stage, while in bovines there are 2633 follicles/cm^2^. They observed that the number of follicles changed with the animal´s age, and at birth it was 1248 follicles/cm^2^. A higher number of hair follicles/cm^2^ would mean that they can help retain a greater amount of heat during exposure to cold, since it would reduce contact with cold air currents, as has been observed with other species where the presence of longer hair and a greater number of hair follicles can serve as a form of defense against the cold [13].

The thickness of the skin and the number of hair follicles participate in maintaining thermoneutrality in the newborn. Another characteristic present in the water buffalo is the number of sweat glands [81]. Debbarma et al. [81] found that sweat glands were located deep in the reticular dermis and observed that the secretory portion was made up of glandular tubules, myoepithelium, and a basal membrane. In addition, they indicated that the ventral abdominal region contained 0.38 ± 0.17 fewer glands/mm^2^ and that this was significantly lower (*p* < 0.05) than the number found in the dorsal and lateral areas (0.98 ± 0.17 and 1.08 ± 0.08 glands/mm^2^, respectively). This reaffirmed what had been previously observed, that there is an average density of 394 sweat glands per cm^2^ in the water buffalo compared to 2633 glands in the domestic bovine [76]. The low presence of sweat glands may directly affect sensitivity to thermal stress due to heat, since it would have a limited capacity to dissipate heat through their evaporation [12,14,82], but can this characteristic influence the thermoregulatory capacity of a newborn in the cold? In this regard, Shafie and Abou El-Khair [83] reported that the secretory activity of the glands in terms of sebum production in the buffalo is higher (111 mg/m^2^) compared to bovines of the Friesian, Shorthorn, and Egyptian breeds (40, 29, 25 mg/m^2^). The air temperature determines this variation, therefore, in the winter months, and when the temperature reaches 13.8 °C, production ranges from 124 ± 30 to 153 ± 35 mg/m^2^. Likewise, it was observed that sebum secretion per skin unit is lower in 6-month-old buffaloes when the air temperature is 27.1 °C compared to a lower temperature (13.8 to 20.8 °C), in which case the secretory activity increases 94 ± 22 mg/m^2^. This secretory action prevents water absorption from a similar surface and allows it to adapt to low thermal conditions and conserve heat. Perhaps these mechanisms favor the reduction of heat loss in the water buffalo, since the phenomena of convection and conduction of cold air intervene when the animal is exposed to a cold environmental temperature [84], such that it is possible to support the idea that the skin characteristics of the newborn buffalo have a higher threshold for cold thermal stress, compared to other species. However, it is not yet clear if these characteristics work to avoid heat loss due to less heat conduction or less heat evaporation, which could lead to ensuring that the newborn water buffalo has cold resilience compared to other species.

### 3.3. Neonatal Vitality

Neonatal vitality depends on birthweight, adipose tissue, glycogen reserves at birth, and immediate colostrum intake contributing to the ruminant newborn’s vitality [85,86,87]. Mülling [88] adapted the first APGAR score in calves to evaluate muscle tone and movement, reflex activity, respiration, and mucous membrane color, classifying them as healthy (7–8 points), at-risk (4–6), or weak (less than 3) [89]. Although these parameters did not consider the temperature at birth, perinatal hypothermia is directly related to vitality and mortality since acidosis –a consequence of fetal stress and dystocia– impairs thermogenesis [90]. Moreover, external factors (e.g., exposure to cold, wet, and windy environments) increase heat loss and decrease the ability of newborns to adapt to the extrauterine environment [2,59,91], as reported by Khounsy et al. [24] in northern and central Lao where temperatures around 6.7–7.5 °C caused the death of 3744 buffaloes.

Vitality is essential because it is closely related to neonatal mortality in the first four weeks of life [89,92]. In the case of water buffalo, studies have reported a mortality rate of 17.9–39.8% [6,93], and according to Mee [90], Holstein-Friesian calves whose rectal temperature at birth decreases from 39–39.5 °C to 38.5–39 °C during the first hour of life are considered as having good vitality, while a temperature drop from 39.5–40 °C to less than 38.5 °C is associated with poor vitality individuals. Likewise, Kozat [1] states that *Bos* calves with body temperatures lower than 35 °C are considered hypothermic and require immediate treatment.

**Figure 2:**
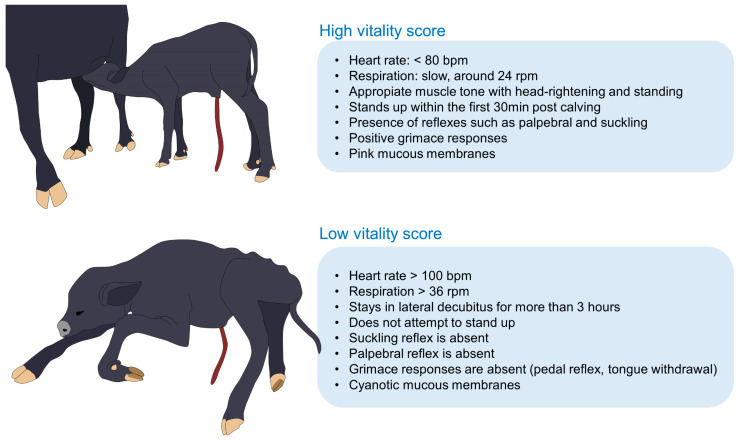
Neonatal vitality parameters in ruminants.

In the case of water buffalo, there are no vitality scores applied during calving or to evaluate the fetus’s health, as was mentioned by Szenci [89] for *Bos* calves. However, current clinical scores designed to assess vitality in ruminants consider physiological parameters such as rectal temperature (RT), heart rate (HR), respiratory function, mucous membrane color, grimace responses (including head shake, corneal reflex, suckling reflex, tongue withdrawal, and pedal reflex), and hair coat appearance (e.g., grade of meconium stain) [89,92]. Likewise, latency to attain sternal recumbency, head righting, and standing up are relevant behaviors that lessen temperature drop (Figure 2) [89,92].

Low vitality scores associated with thermoregulatory issues could be due to the type of birth, whether eutocia or dystocia. This element influences the neonatal physiological, endocrine, and metabolic response during the first hours post-calving [94], as stated by Vannucchi et al. [95], whose evaluations used a modified APGAR score in Holstein calves. When comparing eutocic and dystocic calves, although rectal temperatures were similar among both groups (39.1 °C vs. 39.4 °C, respectively), the vitality score of dystocic newborns was lower (7.2 vs. 8.5) and the blood gas variables were affected, resulting in neonatal acidosis. The same reaction was reported in Sant Ines lambs immediately after lambing and 1 h after. In these animals, Vannucchi et al. [96] found that even eutocic animals presented hypoglycemia and acidosis immediately after lambing.

During the normal course of calving, the metabolic and physiologic outcomes can be partiality addressed when a eutocic newborn can stand up and consume adequate quantities of colostrum [96]. However, low-vitality newborns present an abnormal behavioral repertoire that impedes colostrum intake and other behaviors that prevent hypothermia [95]. In this regard, Murray and Leslie [97] mention that low-vitality neonates cannot ingest colostrum for energy uptake and thermoregulation due to a lack of vigor and energy to stand up and get close to the udder.

If the latency of standing increases, or if low-vitality newborns delay in making the first attempts to stand up, conductive heat loss to the ground continues—along with a combination of convective and evaporative heat losses due to their wet coat—which further impedes attempts at suckling to ingest colostrum and start thermogenesis [85]. In Mediterranean buffalo calves, Lanzoni et al. [98] reported a mean latency to stand up of 77.0 ± 47.5 min. The latency is related to dystocia, as studied by Singh et al. [99] in buffalo calves, where animals delivered through forced calving, in contrast to a normal birth, had a late first attempt to stand up (55.2 ± 9.6 min vs. 31.7 ± 5.4 min, respectively).

Diesch et al. [100] evaluated Angus and Friesian calves at birth to find an association between environmental temperatures and the time to stand, among other parameters such as rectal temperature, lactate, glucose, and fructose concentrations. The authors found that Friesian calves born in climates with air temperatures below 10 °C had lower rectal values (38.6 °C, vs. 39.0 °C in animals exposed to air temperatures above 10 °C) and a delayed standing up (66 min vs. 46 min). Likewise, neonatal hypothermia is also associated with an absent suckle reflex in ruminants, delaying the passive transfer of immunoglobulins and impeding the consumption of energy reserves to produce heat [25]. In water buffaloes, the first suckling occurs at an average of 212.0 ± 110.0 min and can last around 38.0 ± 22.6 min [98]. In general, ungulates start suckling within 90 min after calving [46]. Thus, a longer latency than the reported would suggest that low vitality associated with dystocia, low birth weight, and hypothermia interferes with critical behaviors aimed at thermoregulating (e.g., standing up and colostrum intake) [99]. In addition, Haughey [101] reported cold injuries in newborn Merino lambs reared at 1 °C, which animals have extensive catabolism of fat deposits regardless of colostrum intake. This is relevant in lambs because most of the fat reserves used to thermoregulate in newborns are derived from brown adipose tissue [102].

Therefore, calves with low vitality cannot produce heat as efficiently as a normal newborn, and the effect of hypothermia on the delayed response to ingesting colostrum increases neonatal energy depletion and the risk of mortality [97]. Although some conclusions could be inferred, such as the importance of evaluating the rectal temperature of newborns, some authors have referred to the importance of evaluating physiological data with modified APGAR scores [95], particularly in water buffalo, in whom vitality assessment at birth is limited.

## 4. Effect of Environmental Factors

The environment is among the factors that impact the body temperature in the newborn. In other words, the environmental temperature decreases the body temperature of the water buffalo. This has probably become evident from what has been reported by authors such as Screedhar et al. [103], who observed a decrease in the percentage of neonatal calf mortality during the summer (18.2%) and autumn (16.5%) compared to winter (23.8%) and spring (32.7%). This could be explained by the presence of cold wind, rain, and the drop in environmental temperature that influence rapid heat loss in animals, which increases susceptibility to hypothermia, even though these factors are also associated with respiratory tract diseases. In agreement with this, Torell et al. [104] suggested that the specific etiology of hypothermia in water buffalo is gradual exposure to a cold environment characterized by the constant loss of body heat through respiration but mainly by evaporation of fluid. This condition is described as affecting mainly the youngest members, due to poor body condition or characteristics such as immature thermoregulation systems; for this reason, the use of facilities that avoid the exposure of offspring to a harsh environment was suggested [105].

The temperature and wind speed are some of the variables that significantly influence the body temperature of animals. It has been previously pointed out that, in addition to body temperature, the variables of heart rate and respiratory rate can also be modified according to wind speed and environmental temperature [106]. An example of this was reported by Zhao et al. [107], who evaluated the impact of exposure to cold air with different flows on the response of physiological parameters in 47 Holstein cattle aged 3 to 29 days. They observed that 3-day-old animals exposed to high air velocity (1 m/s) had a 0.13 °C lower rectal temperature and 2.33 fewer breaths per minute than animals exposed to a lower air velocity (4 m/s). However, in 19-day-old animals, this effect was reversed, and they observed that rectal temperature increased by 0.20 °C and breaths per minute by 10 in animals exposed to high airflow. The speed of the wind does have a direct effect on the modulation of physiological parameters. This is explained by the fact that air movement affects the heat exchange rate by convection and evaporation, but it can be limited due to the vasomotor mechanisms that reduce this exchange rate and by less proportional participation of evaporation heat loss. However, if the speed increases (6 m/s), an additional increase in convective heat transfer is generated, leading to heat loss [108,109]. Therefore, the combined effect of wind speed and environmental temperature can accelerate heat loss in the newborn, which has immature thermoregulation mechanisms compared to what is observed in older animals.

On the other hand, the presence of rain or snow combined with a low ambient temperature may also influence the induction of hypothermia in the newborn, with a decrease up to 1.5 °C in body temperature, and an increase in the respiratory rate as a way of compensating for the progressive decrease in body temperature [22,110,111]. The environmental humidity combined with a low temperature can produce hypothermia, increasing the mortality of the newborn. In fact, precipitation can induce hypothermia because water can accumulate in the animal’s fur, displacing still air and reducing insulation from the environment by the hair. This is because rain can flatten the fur and increase its thermal conductivity coefficient, which increases heat loss by conduction as well by convection and later by evaporation [112].

Therefore, the environment should be considered as one of the main factors that affects the body temperature of the newborn buffalo, especially in the case of exposure to wind and the presence of rain. This would make it difficult for the newborn to maintain constant internal heat production so that its body temperature is comparable to the environment´s temperature and thus achieve thermoneutrality.

## 5. Thermoregulatory Mechanisms Triggered to Face Neonatal Hypothermia

**Figure 3:**
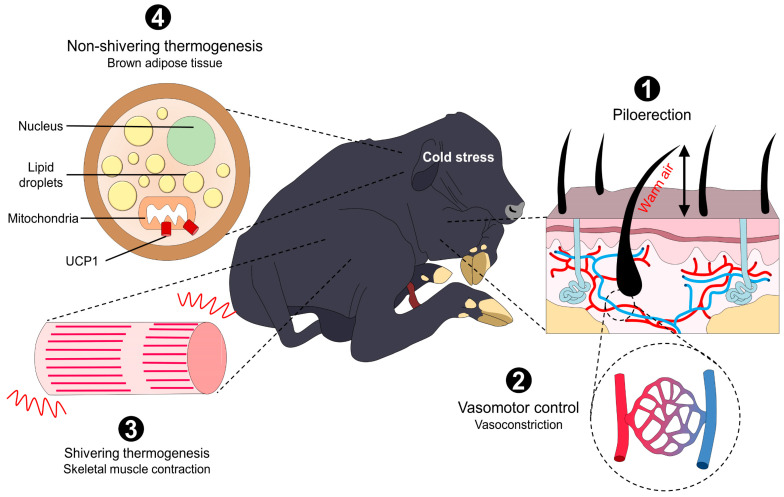
Thermoregulation mechanisms that are activated in response to cold stress. In general, when mammals face acute cold, a sequence of events begins whose purpose is to preserve heat or produce it. In the first instance, vasomotor control (vasoconstriction) and piloerection are mechanisms at the skin level to preserve heat. Thermogenesis by shivering or by using adipose tissue is considered a second mechanism for producing heat when the body is unable to control heat loss. UCP1: uncoupling protein 1.

To achieve thermostability and cope with the several individual and environmental challenges that newborns are exposed to, the activation of metabolic, vasomotor, and muscular activity is required to prevent heat losses [95]. The hypothalamus has a critical role during neonatal hypothermia because it recognizes this situation and triggers said mechanisms (e.g., vasoconstriction, skeletal muscle shivering, and non-shivering usage of brown adipose tissue (BAT)) to maintain and produce heat [2,11]. However, it has been reported that newborn ruminants are born with an underdeveloped hypothalamic–pituitary–adrenal (HPA) axis, making them prone to hypothermia because the thermoregulatory center, the preoptic area (POA), is located in the hypothalamus (Figure 3) [113].

The activation of hypothalamic neurons and their projections to muscle fibers, BAT, or the vascular endothelium requires the participation of peripheral thermoreceptors that detect cold environmental temperatures or critical decreases in core temperature [71]. The so-called cold-sensitive neurons mainly comprise transient receptor potential (TRP) channels from the ankyrin (TRPA1) and melastatin subfamily (TRPM8) [114]. In particular, TRPM8 has a threshold temperature of 28 °C, while TRPA1 activates with noxious cold (28–8 °C) [115].

The receptors that respond to noxious cold, known as nociceptors, are Aδ- and C-fibers that transduce and transmit the thermal signal from the periphery to the POA and another relevant thermoregulatory nucleus, such as the dorsomedial hypothalamus (DMH), via the dorsal lateral parabrachial nucleus (LPB) in the pons, the rostral raphe pallidus (rRPA), and its connection to the spinal cord and motor or sympathetic neurons [116,117].

### 5.1. Vasomotor Control

Vasoconstriction occurs as a response to core temperature or when peripheral thermoreceptors detect cold environmental temperatures [11,118]. This heat-preserving mechanism is triggered by sympathetic neurons that restrict the blood flow from peripheral dermal vessels in regions such as limbs, nose, or auricular pavilion to reduce heat transfer [119,120]. In this sense, skin characteristics, superficial arteries, veins, capillaries, and arteriovenous anastomosis play an essential role in modulating this response and changing the flow rate of the blood [13]. In the case of adult water buffaloes, their skin is thicker than that of *Bos taurus* (an average of 6.03 ± 1.16 mm) [121], increasing the distance between skin blood vessels and the epidermis, which could confer a type of insulator property to make buffaloes resistant to cold temperatures [122]. Nonetheless, as the authors are aware, there are no published studies regarding these characteristics in newborn buffaloes.

When exposed to cold stress, mammals release norepinephrine and neuropeptide Y from sympathetic fibers, as a result of the activation of the HPA [123]. Both mediators bind and activate α1 and α2-receptors to cause vasoconstriction of cutaneous arterioles [120]. This was studied by Slee [124] in Scottish Blackface and Merino sheep exposed to −15 °C or to −5 °C. Intense vasoconstriction was recorded at both temperatures. Particularly, skin temperature (measured with thermocouples) of the feet and ears decreased significantly in both Blackface (from approximately 33 °C to 4 °C and 37 °C to 4 °C, respectively) and Merino animals (from approximately 33 °C to 26 °C and from 37 °C to 15 °C, respectively). The retarded vasoconstriction observed in Merino animals might be influenced by the prominent fleece of the breed, which can serve as a thermal insulator, and suggests that other factors such as the presence of hair, glabrous skin, or coat characteristics also affect the degree of thermoregulatory change. Although there are no studies focusing on vasomotor activity in water buffalo, Turnpenny et al. [125] mention that, in bovine and ovine, vasomotor control is mainly observed on the legs and trunk. However, anatomical regions with glabrous skin have little to no defense against cold temperatures and the amount of radiated heat from these zones could be more easily affected and perceived than in central regions such as the inguinal region [19].

Although vasoconstriction is the first cold-induced autonomous response, other thermogenic and heat-preserving mechanisms can also be activated by newborn buffaloes.

### 5.2. BAT Thermogenesis

**Figure 4:**
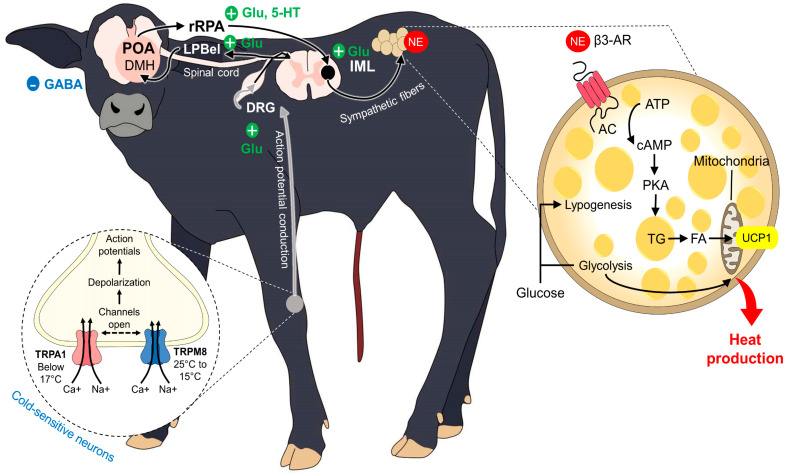
Thermogenesis by activation of brown adipose tissue. When thermoreceptors located in the periphery of newborns detect decreases in temperature (mainly TRPA1 and TRPM8), these cold-sensitive neurons transduce and transmit the thermal signal to the DRG of the spinal cord. Through the LPBel, the signal is in the thermoregulatory center (POA), from which sympathetic neurons are directed towards the IML and towards the BAT. In this adipose tissue, the interaction of NE (released by sympathetic neurons) generates lipogenesis and heat production in the BAT mitochondria, thanks to the activation of UCP1 receptors. AC: adenyl cyclase; β3-AR: beta-3 adrenergic receptor; cAMP: cyclic AMP; DMH: dorsomedial hypothalamus; DRG: dorsal root ganglion; FA: fatty acids; Glu: glutamate; LPBel: external lateral part of the lateral parabrachial nucleus; NE: norepinephrine; PKA: protein kinase A; POA: preoptic area; rRPA: rostral raphe pallidus; TRPA: transient receptor potential ankyrin 1; TRPM8: transient potential receptor melastatin 8. TG: triglycerides; UCP1: uncoupling protein 1.

Non-shivering thermogenesis or BAT thermogenesis is an optional method of rapid heat production in cold environments (Figure 4) [62,126]. It uses energy resources by increasing the basal metabolic rate, which helps increase heat production. BAT differs morphologically and metabolically from white adipose tissue. For example, in sheep during the fetal stage, it appears in the perirenal region at 70 days of gestation [127,128]. Among the various species of mammals, the amount of BAT differs from 8–24 g, as well as the triglyceride content, which can range from 0.40 to 0.80 mg [62,129]. It should be noted that BAT is a tissue that is not present in all species, such as pigs [130] and wild boars (*Sus scrofa*) [131].

BAT provides great energetic value for the newborn because it is used for increasing body temperature. However, in some species, the efficiency of this mechanism is questioned, for example, in the case of the water buffalo. In this regard, Louveau et al. [132] mention that brown adipose tissue occurs more frequently in the perirenal region in large ruminants, but in sheep, BAT deposits are located mainly in the scapular (*regio scapularis*), neck (*collum regionis*), and cardiac (*regio cardiaca*) regions and perirenal fat. In the case of newborn reindeer, brown adipose tissue is found in greater proportion in the perirenal–abdominal region, occupying 32% of the total [133]. Following the above, the hypothesis could be advanced that there are certain species whose thermogenesis mechanisms are more efficient, either because of their amounts of BAT or because of their neurodevelopment.

In some species, the amount of available BAT at birth is lower, making them susceptible to starvation that consequently will lead to states of acute hypothermia [134]. However, there is no doubt that BAT thermogenesis represents the first source of energy in the postnatal period in almost all species, except those that lack large BAT stores, which depend instead mainly on the timely ingestion of colostrum [2]. It is worth mentioning that when talking about unconventional productive species, such as the water buffalo, the scientific evidence is scarce, so it is even more important to understand if the thermoregulation mechanisms in these animals are less efficient given particular anatomical characteristics, such as thicker skin and lower density of hair follicles, which could lead to decreased heat loss [12,14,84].

### 5.3. Shivering Thermogenesis

**Figure 5:**
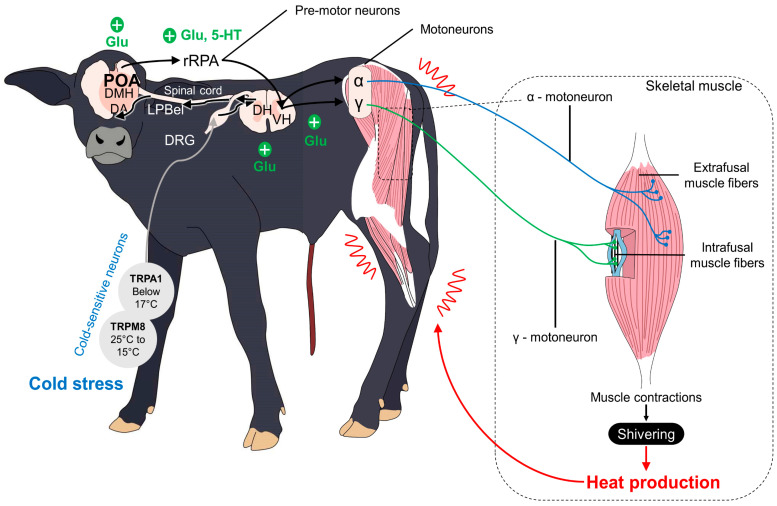
Shivering thermogenesis. Shivering, caused by skeletal muscle contractions in response to activation of α and γ motor neurons, is initiated by activation of cold-sensing neurons. These transmit the information to higher centers such as the DMH and the DA at the POA. Through projections from the POA to the rRPA and VH, motoneurons generate repeated contractions that lead to heat production. DA: dorsal hypothalamic area; DH: dorsal horn; DMH: dorsomedial hypothalamus; DRG: dorsal root ganglion; Glu: glutamate; LPBel: external lateral part of the lateral parabrachial nucleus; POA: preoptic area; rRPA: rostral raphe pallidus; TRPA: transient receptor potential ankyrin 1; TRPM8: transient receptor potential melastatin 8; VH: ventral horn.

Muscular shivering or tremor is a mechanism through which heat can be generated from the rapid and constant movements of the musculoskeletal system (Figure 5) [135]. These movements or contractions generate heat through the oxidation of various substances, among which we can cite lipids, carbohydrates, and proteins, that the individual obtains either through the contribution of food (through the bloodstream) or the same muscle in question [136,137,138].

The degree of muscular development [139] and the quantity of muscle mass that some species have at birth [2] explain why this mechanism can be carried out efficiently in some species and not in others; thus, birth weight could be a factor that must be considered [3,56]. Other factors that directly influence whether shivering can be carried out effectively are the percentage of body fat and the amount of ATP that the species in question requires to generate these muscle contractions [138], and indirectly, we could even mention the quantity and quality of maternal care, since if this behavior does not exist on the part of the mother in an adequate way, the newborn will not receive an adequate energy supply through colostrum and milk [54,140]. There are species in which shivering is not one of the best mechanisms that the newborn has, as in the case of newborn puppies [141], who have it either poorly or not at all developed. In pigs [142,143,144,145] and lambs (at least during the first few days of life) [146], shivering is an important mechanism to counteract hypothermia; however, it is not the first choice in ruminants [63]. According to Smith and Carstens [147], in lambs, BAT thermogenesis is one of the most used mechanisms in the early postnatal period. Due to all of the above, neonatal monitoring and evaluation is necessary to minimize the risk of mortality, since these adaptations help provide a stable and functional thermoregulatory mechanism until after the first four weeks of life [148], especially in species such as the water buffalo, for which there is little research in this regard to this day.

### 5.4. Changes in Behavior and Posture in the Face of Cold Stress

Since hypothermia is a severe problem that newborns of any species have to face, the body makes use of not only the mechanisms described above, but also uses certain behavioral or even postural changes that help it counteract this situation, thereby reducing heat loss and managing to support metabolic mechanisms in a certain way [79]. 

To enumerate some behavioral mechanisms that occur in altricial or semi-altricial species, we could mention strategies such as snuggling with the other individuals of the litter in the case of rabbits [149,150], rats [151], puppies [2,152,153], and pigs [154]; the elaboration of a nest made by the mother as part of maternal care [155]; and learned conditioned behaviors as in the case of laboratory rats [151].

**Figure 6:**
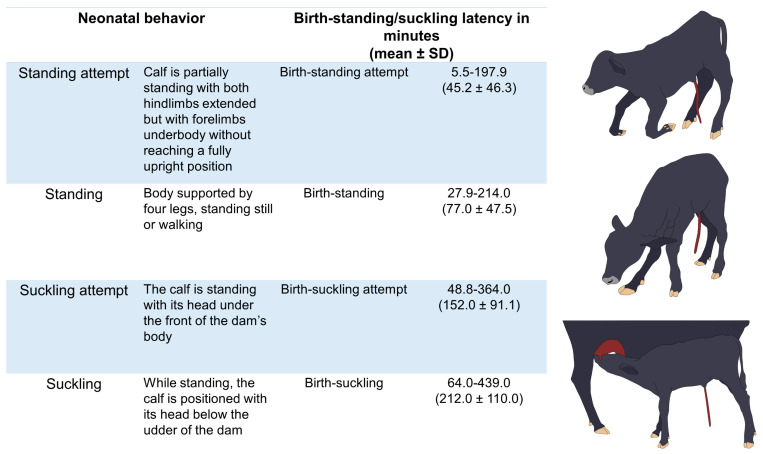
Neonatal behavior and standing/suckling latencies. SD: standard deviation.

In the case of precocial species, such as sheep and other ruminants, standing up immediately after birth is not only functional for the calf to reach the mother’s teat, but also helps in a certain way to avoid having the entire body surface in contact with the ground and thus limits conductive body heat loss [156,157]. Latency times for standing up and ingesting colostrum are schematized in Figure 6 [98].

## 6. Colostrum Intake: Nutritional, Immunological, and Physicochemical Aspects

Colostrum intake has an essential thermoregulatory function for neonates due to its highly energetic nutritional content (11.31 ± 0.39% of fat) [158], which participates in fatty acid oxidation for gluconeogenesis [159]. In this way, colostrum is the main source of energy, nutrients, and immunity for the neonate in the first hours of life [61,130,160], as well as being a source of vitamins and minerals [161]. Colostrum has around 11.37% fat [10,162], together with high concentrations of butyric, myristic, palmitic, and oleic acids [158]. Moreover, colostrum has an Ig distribution of 85% IgG, 8% IgM, and 5% IgA [163]. Colostrum production occurs during the peripartum, beginning 3 to 4 weeks before calving and ending abruptly during calving. During this process, immunoglobulins are transported to the mammary gland (MG), where epithelial receptors enable protein transfer [164,165].

Inadequate provision of colostrum during the immediate post-calving period has serious repercussions on ruminant newborns, such as susceptibility to pathologies in calves, lambs, and goats [10,166]. This susceptibility is due to the placental structure that impairs fetal immunoglobulin transfer, resulting in agammaglobulinemic newborn ruminants [167]. This directly impacts the mortality rates of the calves and the productive and profitability parameters [4,61]. Regarding colostrum intake, Mellor and Murray [168] determined that the amount highly depends on the birth weight. In lambs, it is recommended to provide 180 and 210 mL/kg during the first 6 to 18 h of life; in cattle, 5% of birth weight is suggested to ensure passive immunity in the newborn [169].

In comparison to milk, colostrum has higher percentages of protein, vitamins, minerals, peptides, carbohydrates, hormones, and cytokines. For example, colostrum protein content is 6.9% higher than mature milk in Murrah dams [170]. In Egyptian buffaloes, 12.94% protein in colostrum has been reported [171], 10.65% in Nili-Ravi buffaloes [172], and 8.73% in Italian buffaloes [158], represented by immunoglobulins (mainly IgG, IgM, and IgA [173]), β-lactoglobulin, and α-lactalbumin [158]. Regarding mineral content, in Nili-Ravi buffaloes, high values of Ca, Mg, Na, K, and Fe are observed (2132 mg/kg, 187 mg/kg, 692 mg/kg, 1813 mg/kg, and 2.81 mg/kg, respectively), values that decrease as mature milk is secreted by MG [172]. However, the quality of minerals and other components can be influenced by maternal factors such as parity. This was reported by An et al. [174] when comparing multiparous and primiparous buffalo dams, where multiparous animals had higher concentration of Fe, Na, Mg, Co, and K (*p* < 0.05), and better IgG levels (39.85 vs. 35.25 mg/mL).

On the other hand, since ruminant newborns develop active immunity after three weeks of life, the passive transfer of antibodies through colostrum is necessary during the first 24 to 36 h after calving. It has been reported that the absorption efficiency of immunoglobulins decreases from 66% at 6 h after birth to 50% at 12 h [10,175]. Barmaiya et al. [175] evaluated 24 ninety-day-old calves and 24 newborn buffaloes, finding that IgM, IgG, and IgA immunoglobulins in serum samples increase in older calves and after colostrum feeding, particularly IgG and IgA. Additionally, colostrum is also associated with growth, as mentioned by Erdem et al. [176], who evaluated the relation between the specific gravity of colostrum and the growth and development of Anatolian calves. Classifying the specific gravity either 1 or 2 (<1.070 and ≥1.070 g/mL respectively), the authors found that individuals from the second group had higher chest circumference, height at the withers, and body weight than group 1.

The quality of this product is influenced by factors such as breed, age of the mother, number of parity, duration of the dry period, milk yield, time of calving, nutritional status, and live weight, among others [167]. In this sense, Silva et al. [177] evaluated the thermal response of 30 newborn Holstein calves subjected to a thermal challenge (10 °C) for 150 min and fed at 2 and 8 h of life with different volumes of high-quality colostrum according to birth weight. According to surface and rectal temperature, the authors concluded that provision of 15 and 20% of colostrum resulted in higher rectal temperatures (15% had 38.1 °C and 20% had 38 °C). The thermal changes between groups of calves can be associated with increased colostrum consumption and development in the absorption and degradation capacity of triglycerides used for heat production [97,177].

Colostrum properties improve neonatal survival and might decrease mortality associated with hypothermia because it is a source of energy for the calf [25,176]. Therefore, it is essential to provide colostrum within the critical period of 36 h post calving (maximum), and it is also relevant to adopt monitoring techniques to ensure a proper thermal state of the newborn [11,25,178].

## 7. Evaluation of the Thermal Response through Infrared Thermography (IRT)

The temperature changes and the physio-metabolic consequences that newborn buffalo suffer due to hypothermia require a method to address heat loss early. For this reason, IRT has been suggested as a way to assess the thermal response in newborns [25,32]. The non-invasiveness of IRT permits a real-time assessment [15] that has shown a strong correlation with body temperature [179,180]. However, since the thermal response depends on the anatomical site and the vasomotor response, it is important to discuss the different regions that can be used to assess the thermal response of newborns.

### 7.1. Central Thermal Windows

**Figure 7:**
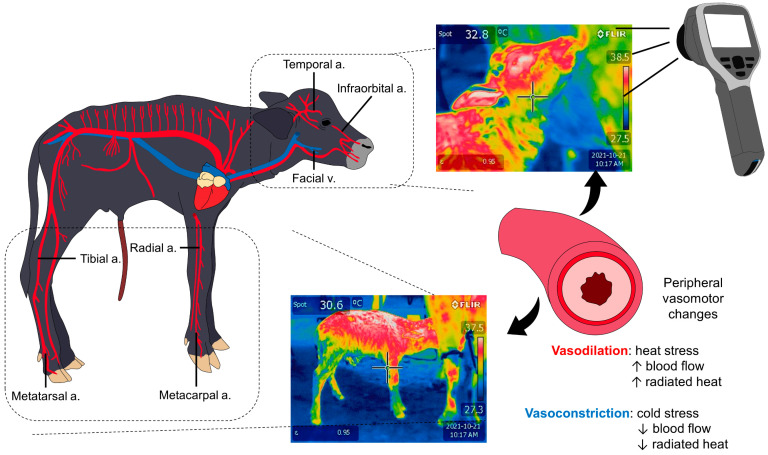
Vascularization of the newborn ruminant and its relationship with infrared thermography. Infrared thermography, as a technique that detects changes in blood flow, depends on the circulatory system of animals and the changes associated with the perception of cold.

IRT detects the superficial thermal response by identifying changes in heat exchange in anatomical regions with large densities of blood capillaries and arteriovenous anastomoses, known as thermal windows [19]. These thermal windows participate in heat gain and loss by modifying the vasomotor response. For example, when exposed to cold, vasoconstriction of skin blood vessels reduces the amount of irradiated heat in peripheral structures to redistribute the blood flow to central regions (Figure 7) [181]. Different authors have described the ocular and nasal windows as central thermal windows, although the physiological responses differ [17,18,19,182].

The thermal window of the nasal region has been suggested to assess the respiratory rate (RR). In pigs, the temperature of the nasal region is positively correlated (r = 0.8) with RR [183]. Similarly, in cattle, Stewart et al. [184] used IRT and accelerometry in twenty-two 5.1-year-old cows (Friesian and Friesian x Jersey breed) to evaluate the RR and the response to exercise of the pelvic limbs. The thermal response of the nasal window was able to predict changes in the respiratory pattern. Similar to these results, Lowe et al. [185] reported a high correlation (r^2^ = 0.92) of the conventional method of counting flank movements in the respiratory cycle with thermal changes around the nostrils in 5 Hereford calves of 27 ± 3.7 days old. Based on these results, the temperature of the nasal region could indirectly indicate RR in a non-invasive way in these animals, in which, due to their size and handling, it may be challenging to evaluate this parameter.

Regarding the association between the nasal temperature and hypothermia in neonates, Lezama García et al. [56] suggested that the temperature of the nasal and thoracic region was 3.1 °C and 6 °C higher than the temperature of the pelvic limb in newborn dog pups. The differences between both regions could be understandable by what is observed in the ocular thermal window. In this context, Shu et al. [186] evaluated 40 Holstein lactating calves that were exposed to heat stress. These authors observed that the temperature of the ocular region and respiratory rate were significantly correlated (r^2^ = 0.55, *p* < 0.05). So, when an animal is exposed to a low environmental temperature, a compensatory mechanism modulates the blood flow as a result of the activation of the SNS [25]. Likewise, Sutherland et al. [187] evaluated the ocular temperature based on the activity of the autonomic nervous system (ANS) in 20 Romney sheep. It was observed that the maximum ocular temperature was 0.5 °C higher in the animals that received epinephrine. Since hypothermia can be considered a stressor that activates the SNS, catecholamine release is also an event present in newborns exposed to cold temperatures. Therefore, evaluating the response of the ocular surface could be considered an indicator of sympathetic activity.

**Figure 8:**
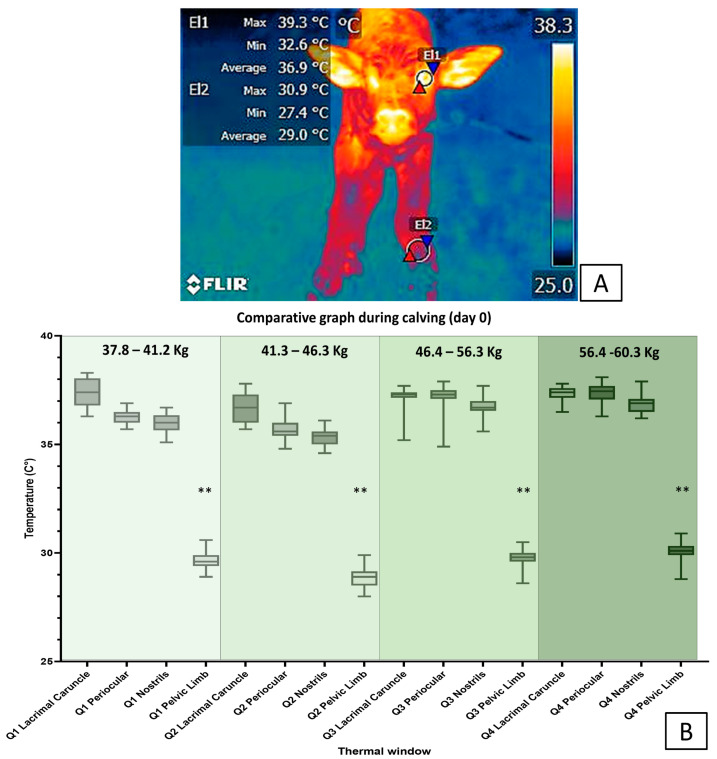
Thermal response in newborn water buffalo with different birth weights and different thermal windows at day 0. (**A**) the periocular thermal window (El1) and the pelvic limb thermal window (El2) can be seen. (**B**) shows the temperature values in different thermal windows according to the weight of the newborn buffalo divided by quartiles on day zero. Maximal temperature is indicated with a red triangle and the minimal with a blue triangle. ** Indicate significant differences between groups (*p* < 0.01).

**Figure 9:**
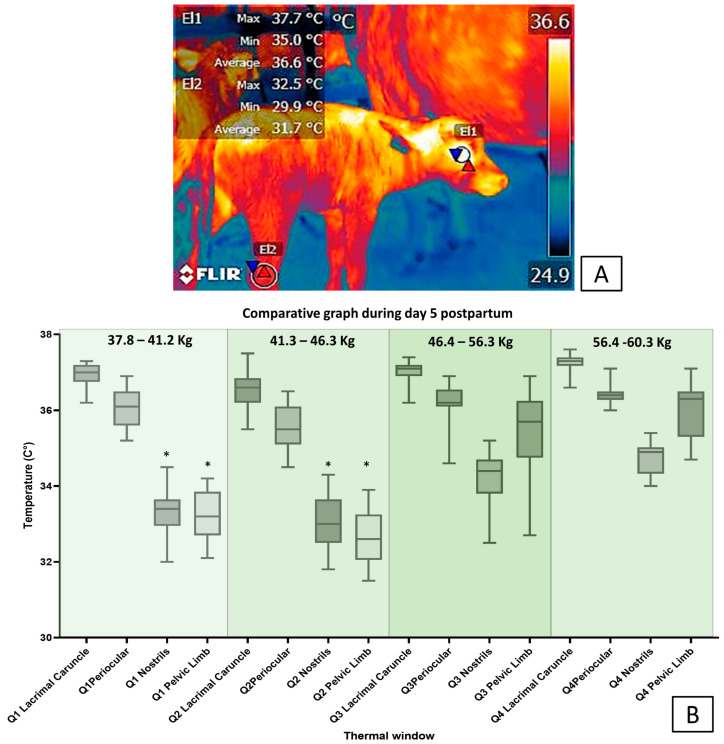
Thermal response in newborn water buffalo with different birth weights and different thermal windows at day 5. (**A**) the surface temperature of the thermal windows in the water buffalo is presented for day 5 postpartum, and it is observed that in the periocular region (El1) it was 36 °C and in the limb pelvic region (El2) it was 31.7 °C. (**B**) shows the temperature values in different thermal windows according to the weight of the newborn buffalo divided by quartiles on day five after birth. Maximal temperature is indicated with a red triangle and the minimal with a blue triangle. * Indicates significant differences between groups (*p* < 0.05).

In this sense, Figure 8 and Figure 9 show that the surface temperatures of the central thermal windows of the eye, and the auricular and nasal pavilions, were significantly higher compared to the peripheral windows in newborn water buffaloes. In Figure 8A, it can be seen from the surface temperature in newborn water buffaloes during the day of delivery, that the periocular thermal window (El1) and the pelvic limb thermal window (El2) presented average temperatures of 36.9 °C and 29 °C, respectively. In Figure 8B, the surface temperature during calving (day 0) showed the trend of the surface temperature of the different thermal windows (lacrimal caruncle, periocular, pelvic limb, and nasal regions) in water buffaloes with different birth weights (Q1 = 37.8–41.2 kg, Q2 = 41.3–46.3 kg, Q3 = 46.4–56.3 kg, and Q4 = 56.4–60.3 kg); examined by means of an ANOVA analysis, it was observed that in general the surface temperature differences of the thermal windows of the lacrimal, periocular, and nasal caruncle, in comparison with the thermal window of the pelvic limb, were statistically significant (*p* < 0.0001), between 5 and 6 °C higher in the four groups of animals of different weight. In Figure 9A, the surface temperature of the thermal windows in the water buffalo is presented for day 5 postpartum, and it is observed that in the periocular region (El1) it was 36 °C and in the limb region pelvic (El2) it was 31.7 °C, the latter increasing its temperature by 2.7 °C. In Figure 9B, it can be seen that the surface temperature in the thermal windows of the lacrimal caruncle and periocular was 4 °C higher than those of the nasal region and pelvic limbs in animals from Q1 and Q2 (*p* < 0.0001). The presence of differences only in animals of lower weight indicates the importance of birth weight to maintain greater thermal stability in the newborn, since these may have greater energy resources that help to recover thermoneutrality, which can be evaluated by IRT. These data were analyzed using an ANOVA test and a Tukey post hoc test in the Prism GraphPad statistical package (ver. 9.7).

The differences being observed only in low-weight animals indicates the importance of birth weight to maintain greater thermal stability in the newborn because they may have greater energy resources. For example, Napolitano et al. [3] evaluated the response in 109 newborn buffaloes of different weights in six thermal windows. The authors found that the temperature in the periocular, nasal, and auricular regions was significantly higher than in peripheral thermal windows in the pelvic limbs (hindlimbs) and thoracic limbs (forelimbs), as expected. Yet, additionally, this thermal response was more efficient in calves with higher birthweights. According to these results, it can be concluded that SNS coordinates the compensatory thermal response during hypothermia and that the newborn’s weight facilitates the maintenance of thermal stability.

**Figure 10:**
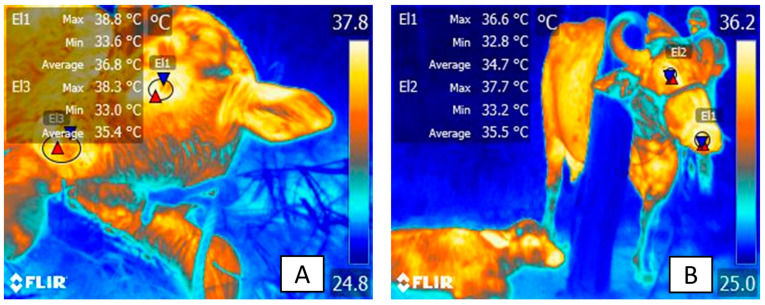
Thermal response of newborn water buffalo calf and postpartum mother. (**A**) Newborn calf. The periocular (El1) and nasal (El3) surface temperatures are presented, where it is possible to observe mean surface temperatures of 36.8 °C and 35.4 °C, respectively; this response may be associated with hypothermia due to the presence of amniotic fluid. (**B**) Postpartum mother. Like the offspring, the thermal response of the periocular (El2) and nasal (El1) regions is presented, where average surface temperatures of 35.5 °C and 34.7 °C, respectively, were presented. From a comparative perspective, it can be observed that the overall surface temperatures of the different thermal windows in the mother were lower by 1.3 °C in the periocular region and 0.7 °C in the nasal region. This could be due to the mother’s perception of acute pain. The activation of the SNS causes the neurosecretion of catecholamines, producing peripheral vasoconstriction, which reduces heat radiation, while this same response of the ANS in the calf allows an increase in temperature due to the different mechanisms of thermogenesis and the vasomotor control of temperature in the central windows. Maximal temperature is indicated with a red triangle and the minimal with a blue triangle. Radiometric images were obtained using a T1020 FLIR thermal camera. Image resolution 1024 × 768; up to 3.1 MP with UltraMax. FLIR Systems, Inc. Wilsonville, OR, USA.

It is worth mentioning that it has been found that in regions with the presence of brown adipose tissue, an increase in surface temperature can be observed due to the increase in energy metabolism in the newborn, which allows triggering heat production through thermogenesis without shivering [2,11]. In fact, Plush et al. [62] observed that in sheep, BAT deposits in the hip region or interscapular region are sites that could provide high energy content to compensate for heat loss caused by exposure to a cold environment. This fact was corroborated in a study conducted by Labeur et al. [188], who evaluated the appropriate method to measure heat loss in newborn lambs under cold challenge, with 14 control lambs and 13 lambs from sheared ewes that were in a cold test for 1 h. In general, they observed that the lambs that came from sheared mothers presented better control of body temperature and had 0.6 °C higher compared to the control group. Similarly, these authors observed that the surface temperature of the pelvis (hip) (*Regiones pelvis*) was 3 °C higher compared to the shoulder (joint) (*Regio articulationis humeri*) region. These results confirmed for the authors the ability of IRT to assess thermoregulatory capacity in the newborn. However, with this tool it is also possible to recognize the increase in heat through the consumption of energy resources such as BAT, which is a key mechanism in the newborn, and comparisons can also be made with the response of adults, as shown in Figure 10.

Therefore, applying IRT and the use of central thermal windows (ocular and nasal) can help in recognizing and detecting hypothermia in the newborn in real time. Moreover, by the use of these thermal windows it is possible to identify the level of ANS activity and relevant physiological parameters that may help determine the physiological compensatory capacity.

### 7.2. Peripheral Thermal Windows

Scientific evidence indicates that central thermal windows are positively correlated with body temperature, so these windows could be useful to monitor thermoregulatory capacity in newborn buffaloes. However, some authors like Bertoni et al. [189] and Mota Rojas et al. [15] have suggested peripheral thermal windows at sites such as the pelvic limbs and thoracic limbs.

In this sense, Lezama-García et al. [60], who evaluated 290 newborn dogs of different weights, observed that the surface temperature of the thoracic and pelvic limb windows presented a lower surface temperature compared to the surface temperature of the thermal windows of the abdominal, thoracic, nasal and palpebral regions. These authors explain that the difference in surface temperature may be due to the fact that, during hypothermia states, there is initially a peripheral vasoconstriction response, and this event allows the reduction of heat loss due to the effects of thermal exchange.

Rowan [63] explains that the vasomotor response that is induced during exposure to cold in the newborn is due to the coordination of the activity of the ANS, which causes the neurosecretion of catecholamines and, with it, generates the vasoconstriction of the superficial capillaries in the dermis. Therefore, due to the decrease in blood circulation in these capillaries, heat exchanges between the skin surface and the environment can be reduced [73,134]. This change in dermal circulation, which occurs due to the response to cold, can be recorded by IRT, which could possibly serve as a means of assessing the thermal response to cold early [32]. In fact, Casas Alvarado et al. [182] suggest that the peripheral thermal windows of the limb region could be considered potentially sensitive to temperature changes due to the presence of arterial projections in the environment; for example, the superficial brachial artery (*arteriae brachialis superficialis*) in the thoracic limb region and the femoral artery (*arteriae femoralis)* in the pelvic limb, can serve as sources of heat loss through changes in the circulation of these blood vessels. Napolitano et al. [3] reported that in the newborn buffalo, the surface temperature is lower in the thermal windows of the pelvic limbs, but, curiously, the temperature of this region increased by up to 6.5 °C in animals with greater weight, compared to an increase of 4.3 °C in animals with less weight.

**Figure 11:**
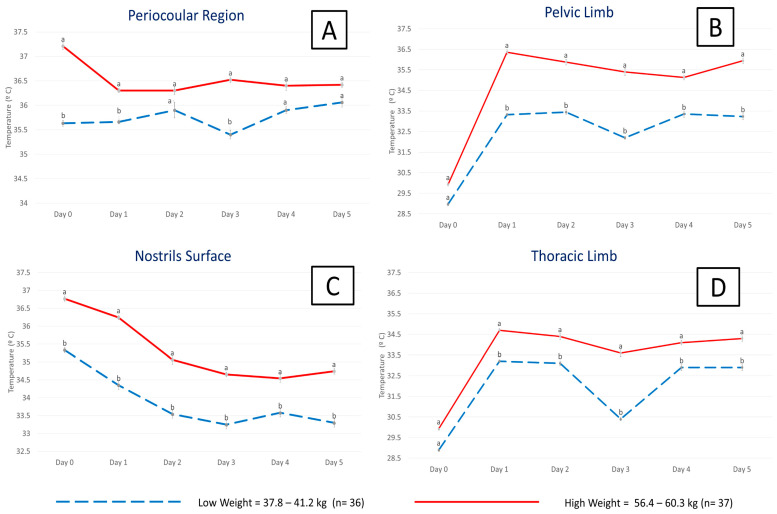
Effect of birth weight on the thermal response of different thermal windows in newborn water buffaloes during the first 5 days of life. Graph (**A**) shows the IRT for the surface temperature of the periocular region thermal window in relation to the weights of the calves on the day of birth. Graph (**B**) shows the IRT for the surface temperature of the thermal window of the pelvic limb. In panel (**C**), the IRT is shown for the surface temperature of the thermal window of the nasal region. In Graph (**D**), the IRT is shown for the surface temperature of the thermal window of the thoracic limb. ^a,b^ Different literals indicate significant differences between groups of high- and low-weight buffalo calves (*p* < 0.05).

This is exemplified in Figure 11, where the results show the importance of the vasomotor thermal response in maintaining heat in central regions such as the thorax and brain region, observed in the region of the auditory canal and lacrimal caruncle. In Figure 11A, the IRT for the surface temperature of the periocular region in relation to the weights on the day of calving, the thermal window shows that during the day of calving in calves with low birth weight, the surface temperatures were 1.5 °C lower compared to calves with high birth weight (*p* = 0.03). It should be mentioned that, in low-weight calves, the temperature gradually increased by up to 0.5 °C by day 5 after birth, which was not the case with high-weight calves.

In Figure 11B, the IRT for the superficial temperature of the thermal window of the pelvic limb, in relation to the weights on the day of calving, shows both groups of calves reached 29–30 °C on the day of birth, which could corroborate the vasomotor response associated with hypothermia. Although the surface temperature increased by 6 °C in the calves with the lowest birth weight, 3 °C lower surface temperatures were recorded in comparison with the high-weight calves (*p* < 0.0001). This response is not significantly observed for the periocular thermal windows.

In Figure 11C, the IRT for the superficial temperature of the nostrils surface thermal window showed a gradual decrease in the superficial temperature of 3.5 °C, which could possibly be explained by the increase in the activity of the SNS, which increases the respiratory rate to increase the availability of O_2_ to recover thermo-neutrality.

In Figure 11D, the IRT for the superficial temperature of the thoracic limb showed that the temperature on day 0 was 4.5 °C lower compared to the rest of the days after birth, both in calves with low weight and in those with high weight (*p* < 0.0001). In addition, a trend similar to that observed in the thermal window of the pelvic limb was observed, where the temperature in low-weight calves was significantly lower compared to high-weight calves. Data analysis was performed using a mixed linear model and a Tukey post hoc test in the Prism GraphPad statistical package (ver. 9.7); letter differences indicate statistical differences between calf weights with a significance level of *p* < 0.05.

This could possibly be explained due to the increased activity of the SNS, which causes a vasoconstriction response that helps maintain thermoneutrality. These preliminary results suggest that the vasomotor and thermoregulation response is related to the energy resources available in the animal. Similarly, according to a review by Gómez-Prado et al. [18], this has been suggested in the case of pigs, and even due to the consumption of colostrum, which makes it possible to increase the thermal response in these regions. In fact, in human medicine, it is reported that the vasomotor response can be canceled or diminished in neonates with low birth weight, and even that the incidence of hypothermia can increase by 6% due to low weight [190]. However, this relationship between birth weight and vasomotor response is still not clear.

Therefore, recordings obtained in the peripheral windows would help to recognize an early vasomotor response to hypothermia to preserve heat. While the central thermal windows, such as the periocular and nasal regions, could indicate the thermal capacity of the animal in hypothermic states, it is necessary to comprehensively assess more than one thermal window to provide complete monitoring of the thermal state in the newborn water buffalo.

## 8. Future Directions

Research trends point mainly to the possible differences in thermoregulation that may exist in the water buffalo. Perhaps one of the clearest examples is the distribution of the BAT, which in lambs is located mainly in the hip and back region [188]. However, there is no clear evidence indicating how this tissue is distributed in the newborn water buffalo, possibly indicating that this thermoregulation mechanism is less efficient or used to a lesser extent in this species. In this regard, it is necessary to establish a relationship between the weight of the animals at birth and how this can affect the primary thermoregulation mechanisms, such that a less effective vasomotor response is obtained, as has been suggested in humans, regardless of the provision of energy resources [190]. At the same time, it is important to determine the correlation with other variables, such as blood and vitality biomarkers [191,192].

Finally, the suggestion of different thermal windows in the bovine species leads to the need to improve the effectiveness of IRT as a tool that can provide important information about the thermal state of the animal [78,79]. Therefore, it is possible that one of the trends in future research will be the application of various thermal windows that provide information about the individual’s temperature in the presence of hypothermia in water buffalo. Several studies have been carried out in this regard in other species, finding that the surface temperatures of the abdominal, lateral, and dorsal thermal windows [81], as well as those of the pelvic area [3,56] or the periocular, can provide information about stressful events [193]. However, more research is needed in this regard in water buffaloes.

## 9. Conclusions

In recent years, water buffaloes have become more popular and they are now being bred in different geographic areas. Therefore, it is essential to deepen the study of this species and carry out research aimed at knowing all their compensatory mechanisms in order to understand and treat hypothermia promptly. Knowledge of their anatomy–physiology is of vital importance to help reduce mortality in this species. Hypothermia is one of the main causes of high mortality rates in newborn animals, both in altricial and precocious species. It is known that, in the case of altricial animals, the mechanisms to compensate for thermal changes are less developed than in precocial species, among which the water buffalo is included. From birth, buffaloes can perform functions similar to adults, such as seeing, hearing, and standing. However, in this species, thermoregulation in animals with hypothermia is difficult to control for newborn buffaloes, due to both morphological and neurodevelopmental differences that have been described throughout this review. For this reason, studying the species in connection with this problem, and carrying out research aimed at understanding its compensatory mechanisms, allows us to understand and attend to the problem appropriately.

In the same way, the study and application of IRT through the various thermal windows used in other species allows the evaluation of thermal changes in water buffaloes, as well as the selection of the most suitable anatomical regions for its application in this species.

## References

[1] Kozat S. (2018). Hypothermia in newborn calves. J. Istanbul Vet. Sci..

[2] Lezama-García K., Mota-Rojas D., Martínez-Burnes J., Villanueva-García D., Domínguez-Oliva A., Gómez-Prado J., Mora-Medina P., Casas-Alvarado A., Olmos-Hernández A., Soto P. (2022). Strategies for hypothermia compensation in altricial and precocial newborn mammals and their monitoring by infrared thermography. Vet. Sci..

[3] Napolitano F., Bragaglio A., Braghieri A., El-Aziz A.H.A., Titto C.G., Villanueva-García D., Mora-Medina P., Pereira A.M.F., Hernández-Avalos I., José-Pérez N. (2023). The effect of birth weight and time of day on the thermal response of newborn water buffalo calves. Front. Vet. Sci..

[4] Rodríguez-González D., Minervino A.H.H., Orihuela A., Bertoni A., Morales-Canela D.A.D.A., Álvarez-Macías A., José-Pérez N., Domínguez-Oliva A., Mota-Rojas D., Hamad A.M. (2022). Handling and physiological aspects of the dual-purpose water buffalo production system in the mexican humid tropics. Animals.

[5] Ahmad S., Yaqoob M., Hashmi N., Zaman M.A., Amjad M.S. (2009). Farmers’ attitude towards interventions regarding buffalo calf health care and management practices under field conditions. Pak. Vet. J..

[6] Khan Z.Z.U., Khan S., Ahmad N., Raziq A. (2007). Investigation of mortality incidence and managemental practices in buffalo calves at commercial dairy farms in Peshawar City. J. Agric. Biol. Sci..

[7] Hancock R.D., Coe A.J., Conde de Albite Silva F. (1996). Perinatal mortality in lambs in Southern Brazil. Trop. Anim. Health Prod..

[8] Hassan N., Shaheen M., Bashir S. (2020). Hypothermia in a Lamb: A case report. J. Entomol. Zool. Stud..

[9] Powers D.R., Langland K.M., Wethington S.M., Powers S.D., Graham C.H., Tobalske B.W. (2017). Hovering in the heat: Effects of environmental temperature on heat regulation in foraging hummingbirds. R. Soc. Open Sci..

[10] Lotito D., Pacifico E., Matuozzo S., Musco N., Iommelli P., Tudisco R., Lombardi P. (2023). Colostrum management for buffalo calves: A review. Vet. Sci..

[11] Mota-Rojas D., Titto C.G., Orihuela A., Martínez-Burnes J., Gómez-Prado J., Torres-Bernal F., Flores-Padilla K., Carvajal-de la Fuente V., Wang D., la Fuente V.C. (2021). Physiological and behavioral mechanisms of thermoregulation in mammals. Animals.

[12] Mota-Rojas D., Napolitano F., Braghieri A., Guerrero-Legarreta I., Bertoni A., Martínez-Burnes J., Cruz-Monterrosa R., Gómez J., Ramírez-Bribiesca E., Barrios-García H. (2021). Thermal biology in river buffalo in the humid tropics: Neurophysiological and behavioral responses assessed by infrared thermography. J. Anim. Behav. Biometeorol..

[13] Mota-Rojas D., Titto C.G., de Mira Geraldo A., Martínez-Burnes J., Gómez J., Hernández-Ávalos I., Casas A., Domínguez A., José N., Bertoni A. (2021). Efficacy and function of feathers, hair, and glabrous skin in the thermoregulation strategies of domestic animals. Animals.

[14] Bertoni A., Napolitano F., Mota-Rojas D., Sabia E., Álvarez-Macías A., Mora-Medina P., Morales-Canela A., Berdugo-Gutiérrez J., Guerrero- Legarreta I., Mendoza A.B. (2020). Similarities and differences between river buffaloes and cattle: Health, physiological, behavioral and productivity aspects. J. Buffalo Sci..

[15] Mota-Rojas D., Martínez-Burnes J., Casas-Alvarado A., Gómez-Prado J., Hernández-Ávalos I., Domínguez-Oliva A., Lezama-García K., Jacome-Romero J., Rodríguez-González D., Pereira A.M.F. (2022). Clinical usefulness of infrared thermography to detect sick animals: Frequent and current cases. CABI Rev..

[16] Schaefer A.L., Cook N., Tessaro S.V., Deregt D., Desroches G., Dubeski P.L., Tong A.K.W., Godson D.L. (2004). Early detection and prediction of infection using infrared thermography. Can. J. Anim. Sci..

[17] Verduzco-Mendoza A., Bueno-Nava A., Wang D., Martínez-Burnes J., Olmos-Hernández A., Casas A., Domínguez A., Mota-Rojas D. (2021). Experimental applications and factors involved in validating thermal windows using infrared thermography to assess the health and thermostability of laboratory animals. Animals.

[18] Gómez-Prado J., Pereira A.M.F., Wang D., Villanueva-García D., Domínguez-Oliva A., Mora-Medina P., Hernández-Avalos I., Martínez-Burnes J., Casas-Alvarado A., Olmos-Hernández A. (2022). Thermoregulation mechanisms and perspectives for validating thermal windows in pigs with hypothermia and hyperthermia: An overview. Front. Vet. Sci..

[19] Mota-Rojas D., Wang D., Titto C.G., Gómez-Prado J., Carvajal-de la Fuente V., Ghezzi M., Boscato-Funes L., Barrios-García H., Torres-Bernal F., Casas-Alvarado A. (2021). Pathophysiology of Fever and Application of Infrared Thermography (IRT) in the Detection of Sick Domestic Animals: Recent Advances. Animals.

[20] Cuttance E., Laven R. (2019). Perinatal mortality risk factors in dairy calves. Vet. J..

[21] Ratanapob N., VanLeeuwen J., McKenna S., Wichtel M., Stryhn H., Rodriguez-Lecompte J.C., Menzies P., Wichtel J. (2020). Management factors associated with perinatal lamb mortality in Prince Edward Island flocks. Prev. Vet. Med..

[22] Slee J., Griffiths R.G., Samson D.E. (1980). Hypothermia in newborn lambs induced by experimental immersion in a water bath and by natural exposure outdoors. Res. Vet. Sci..

[23] Refshauge G., Brien F.D., Hinch G.N., van de Ven R. (2016). Neonatal lamb mortality: Factors associated with the death of Australian lambs. Anim. Prod. Sci..

[24] Khounsy S., Nampanya S., Inthavong P., Yang M., Khamboungheung B., Avery M., Bush R., Rast L., Windsor P.A. (2012). Significant mortality of large ruminants due to hypothermia in northern and central Lao PDR. Trop. Anim. Health Prod..

[25] Mota-Rojas D., Wang D.D.-H., Titto C.G., Martínez-Burnes J., Villanueva-García D., Lezama K., Domínguez A., Hernández-Avalos I., Mora-Medina P., Verduzco A. (2022). Neonatal infrared thermography images in the hypothermic ruminant model: Anatomical-morphological-physiological aspects and mechanisms for thermoregulation. Front. Vet. Sci..

[26] Mota-Rojas D., Bragaglio A., Braghieri A., Napolitano F., Domínguez-Oliva A., Mora-Medina P., Álvarez-Macías A., De Rosa G., Pacelli C., José N. (2022). Dairy buffalo behavior: Calving, imprinting and allosuckling. Animals.

[27] Marai I.F.M., Haeeb A.A.M. (2010). Buffalo’s biological functions as affected by heat stress—A review. Livest. Sci..

[28] Londoño C.R., Sánchez E.N., Prada Sanmiguel G.A.A., Londoño R.C., Sánchez M.E.N., Prada Sanmiguel G.A.A. (2012). Parámetros fisiológicos y valores hematológicos normales en búfalos (*Bubalus bubalis*) del Magdalena Medio colombiano. Rev. Med. Vet..

[29] Asakura H. (2004). Fetal and Neonatal Thermoregulation. J. Nippon Med. Sch..

[30] Rook J.S., Scholman G., Wing-Proctor S., Shea M. (1990). Diagnosis and control of neonatal losses in sheep. Vet. Clin. North Am. Food Anim. Pract..

[31] Menant O., Ungerfeld R., Pérez-Clariget R., Freitas-de-Melo A. (2020). Is body surface temperature measured on the single lambs’ back a reliable indicator of the ewe-lamb bond around birth?. J. Therm. Biol..

[32] Villanueva-García D., Mota-Rojas D., Martínez-Burnes J., Olmos-Hernández A., Mora-Medina P., Salmerón C., Gómez J., Boscato L., Gutiérrez-Pérez O., Cruz V. (2021). Hypothermia in newly born piglets: Mechanisms of thermoregulation and pathophysiology of death. J. Anim. Behav. Biometeorol..

[33] Olson D., Ritter R., Papasian C., Gutenberger S. (1981). Sympathoadrenal and adrenal hormonal responses of newborn calves to hypothermia. Can. J. Comp. Med..

[34] Jain A.K., Tripathi R.K., Sharma I.J., Quadri M.A., Agrawal R.G. (2007). Relationship of serum lipids with development of hypothermia in neonatal bovines. Buffalo Bull..

[35] Mellor D.J., Stafford K.J. (2004). Animal welfare implications of neonatal mortality and morbidity in farm animals. Vet. J..

[36] Tyler H., Ramsey H. (1991). Hypoxia in neonatal calves: Effect on selected metabolic parameters. J. Dairy Sci..

[37] Nuñez A., Benavente I., Blanco D., Boix H., Cabañas F., Chaffanel M., Fernández-Colomer B., Fernández-Lorenzo J.R., Loureiro B., Moral M.T. (2018). Estrés oxidativo en la asfixia perinatal y la encefalopatía hipóxico-isquémica. An. Pediatría.

[38] Marcato F., van den Brand H., Kemp B., van Reenen K. (2018). Evaluating potential biomarkers of health and performance in veal calves. Front. Vet. Sci..

[39] Dubey P., Singh R.R., Choudhary S.S., Verma K.K., Kumar A., Gamit P.M., Dubey S., Prajapati K. (2018). Post parturient neonatal behaviour and their relationship with maternal behaviour score, parity and sex in surti buffaloes. J. Appl. Anim. Res..

[40] Metz J., Metz J.H.M. (1986). Maternal influence on defecation and urination in the newborn calf. Appl. Anim. Behav. Sci..

[41] Jainudeen M., Hafez E., Hafez E., Hafez B. (2000). Cattle and buffalo. Reproduction in Farm Animals.

[42] Lammoglia M.A., Bellows R.A., Grings E.E., Bergman J.W. (1999). Effects of prepartum supplementary fat and muscle hypertrophy genotype on cold tolerance in newborn calves. J. Anim. Sci..

[43] Lehtonen L., Gimeno A., Parra-Llorca A., Vento M. (2017). Early neonatal death: A challenge worldwide. Semin. Fetal Neonatal Med..

[44] Orihuela A., Mota-Rojas D., Strappini A., Serrapica F., Braghieri A., Mora-Medina P., Napolitano F. (2021). Neurophysiological mechanisms of cow–calf bonding in buffalo and other farm animals. Animals.

[45] Mora-Medina P., Napolitano F., Mota-Rojas D., Berdugo-Gutiérrez J., Ruiz-Buitrago J., Guerrero-Legarreta I. (2018). Imprinting, sucking and allosucking behaviors in buffalo calves. J. Buffalo Sci..

[46] Whalin L., Weary D.M., von Keyserlingk M.A.G. (2021). Understanding behavioural development of calves in natural settings to inform calf management. Animals.

[47] Barrier A.C., Ruelle E., Haskell M.J., Dwyer C.M. (2012). Effect of a difficult calving on the vigour of the calf, the onset of maternal behaviour, and some behavioural indicators of pain in the dam. Prev. Vet. Med..

[48] Fitzgerald K.T., Newquist K.L. (2010). Husbandry of the neonate. Small Anim. Pediatr..

[49] Aoki M., Nomura F., Kawata H., Mayer J.E. (1993). Effect of calcium and preischemic hypothermia on recovery of myocardial function after cardioplegic ischemia in neonatal lambs. J. Thorac. Cardiovasc. Surg..

[50] Wood T., Thoresen M. (2015). Physiological responses to hypothermia. Semin. Fetal Neonatal Med..

[51] Thoresen M., Simmonds M., Satas S., Tooley J., Silver I.A. (2001). Effective selective head cooling during posthypoxic hypothermia in newborn piglets. Pediatr. Res..

[52] Martínez-Burnes J., Muns R., Barrios-García H., Villanueva-García D., Domínguez-Oliva A., Mota-Rojas D. (2021). Parturition in Mammals: Animal Models, Pain and Distress. Animals.

[53] Nasr M.A.F. (2017). The effect of stillbirth on reproductive and productive performance of pure Egyptian buffaloes and their crosses with Italian buffaloes. Theriogenology.

[54] Reyes-Sotelo B., Mota-Rojas D., Martínez-Burnes J., Olmos-Hernández A., Hernández-Ávalos I., José N., Casas-Alvarado A., Gómez J., Mora-Medina P. (2021). Thermal homeostasis in the newborn puppy: Behavioral and physiological responses. J. Anim. Behav. Biometeorol..

[55] Reyes-Sotelo B., Mota-Rojas D., Mora-Medina P., Ogi A., Mariti C., Olmos-Hernández A., Martínez-Burnes J., Hernández-Ávalos I., Sánchez-Millán J., Gazzano A. (2021). Blood biomarker profile alterations in newborn canines: Effect of the mother′s weight. Animals.

[56] Lezama-García K., Martínez-Burnes J., Marcet-Rius M., Gazzano A., Olmos-Hernández A., Mora-Medina P., Domínguez-Oliva A., Pereira A.M.F., Hernández-Ávalos I., Baqueiro-Espinosa U. (2022). Is the weight of the newborn puppy related to its thermal balance?. Animals.

[57] Bellows R.A. Factors affecting calving difficulty. Proceedings of the Range Beef Cow Symposium XIII.

[58] Mota-Rojas D., Villanueva-García D., Mota-Reyes A., Orihuela A., Hernández-Ávalos I., Domínguez-Oliva A., Casas-Alvarado A., Flores-Padilla K., Jacome-Romero J., Martínez-Burnes J. (2022). Meconium aspiration syndrome in animal models: Inflammatory process, apoptosis, and surfactant inactivation. Animals.

[59] Mota-Rojas D., López A., Martínez-Burnes J., Muns R., Villanueva-García D., Mora-Medina P., González-Lozano M., Olmos-Hernández A., Ramírez-Necoechea R. (2018). Is vitality assessment important in neonatal animals?. CAB Rev. Perspect. Agric. Vet. Sci. Nutr. Nat. Resour..

[60] Lezama-García K., Martínez-Burnes J., Pérez-Jiménez J.C., Domínguez-Oliva A., Mora-Medina P., Olmos-Hernández A., Hernández-Ávalos I., Mota-Rojas D. (2022). Relation between the dam’s weight on superficial temperature of her puppies at different stages of the post-partum. Vet. Sci..

[61] Napolitano F., Mota-Rojas D., Braghieri A., Guerrero-Legarreta I., Cruz-Monterrosa R.G., José-Pérez N., Álvarez-Macías A., Domínguez-Oliva A., Rodríguez-González D., Lezama-García K. (2022). Colostrum in the water buffalo: Immunological, nutritional and physicochemical aspects. Soc. Rural. Prod. Medio Ambient..

[62] Plush K., Brien F.D., Hebart M.L., Hynd P.I. (2016). Thermogenesis and physiological maturity in neonatal lambs: A unifying concept in lamb survival. Anim. Prod. Sci..

[63] Rowan T.G. (1992). Thermoregulation in neonatal ruminants. BSAP Occas. Publ..

[64] Bienboire-Frosini C., Wang D., Marcet-Rius M., Villanueva-García D., Gazzano A., Domínguez-Oliva A., Olmos-Hernández A., Hernández-Avalos I., Lezama-García K., Verduzco-Mendoza A. (2023). The role of brown adipose tissue and energy metabolism in mammalian thermoregulation during the perinatal period. Animals.

[65] Vázquez-Mandujano E., Reis-de-Souza T.C., Ramírez-Rodríguez E., Mariscal-Landín G. (2019). Impacto del peso al nacimiento del lechón sobre los balances de nitrógeno y energía en la fase de crecimiento. Rev. Mex. Ciencias Pecu..

[66] Barreto J.V.P., Pertile S.F.N., de Almeida Rego F.C., Patelli T.H.C., Nascimento S.T., Lorenzetti E., da Cunha Filho L.F.C. (2021). Prediction of vitality and survival of newborn lambs using a modified Apgar score. Appl. Anim. Behav. Sci..

[67] Dwyer C.M., Morgan C.A. (2006). Maintenance of body temperature in the neonatal lamb: Effects of breed, birth weight, and litter size1. J. Anim. Sci..

[68] Vicente-Pérez R., Avendaño-Reyes L., Correa-Calderón A., Mellado M., Meza-Herrera C.A., Montañez-Valdez O.D., Macías-Cruz U. (2019). Relationships of body surface thermography with core temperature, birth weight and climatic variables in neonatal lambs born during early spring in an arid region. J. Therm. Biol..

[69] Johanson J.M., Berger P.J. (2003). Birth Weight as a predictor of calving ease and perinatal mortality in holstein cattle. J. Dairy Sci..

[70] Nowak R., Poindron P. (2006). From birth to colostrum: Early steps leading to lamb survival. Reprod. Nutr. Dev..

[71] Lezama-García K., Mota-Rojas D., Pereira A.M.F., Martínez-Burnes J., Ghezzi M., Domínguez A., Gómez J., de Mira Geraldo A., Lendez P., Hernández-Ávalos I. (2022). Transient Receptor Potential (TRP) and thermoregulation in animals: Structural biology and neurophysiological aspects. Animals.

[72] Bautista D.M., Siemens J., Glazer J.M., Tsuruda P.R., Basbaum A.I., Stucky C.L., Jordt S.-E., Julius D. (2007). The menthol receptor TRPM8 is the principal detector of environmental cold. Nature.

[73] Romanovsky A.A. (2014). Skin temperature: Its role in thermoregulation. Acta Physiol..

[74] Bhattacharya M., Sheikh I.U., Rajkhowa J. (2003). Epidermal thickness in the skin of Yak (*Poephagus poephagus*). Indian J. Vet. Anat..

[75] Bazzaz A.A. (2020). Poor nociceptive innervation of neck skin in three domestic ruminants. Eur. J. Mol. Clin. Med..

[76] Hafez E.S.E., Badreldin A.L., Shafei M.M. (1955). Skin structure of Egyptian buffaloes and cattle with particular reference to sweat glands. J. Agric. Sci..

[77] Ibrahim R.S., Hussin A.M.H. (2018). Comparative Histological study of the integument in buffallo and cow. Diyala J. Agric. Sci..

[78] Casas-Alvarado A., Mota-Rojas D., Hernández-Ávalos I., Mora-Medina P., Olmos-Hernández A., Verduzco-Mendoza A., Reyes-Sotelo B., Martínez-Burnes J. (2020). Advances in infrared thermography: Surgical aspects, vascular changes, and pain monitoring in veterinary medicine. J. Therm. Biol..

[79] Mota-Rojas D., Pereira M.F.A., Wang D., Martínez-Burnes J., Ghezzi M., Hernández-Ávalos I., Lendez P., Mora-Medina P., Casas A., Olmos-Hernández A. (2021). Clinical applications and factors involved in validating thermal windows in large rumiants to assess health and productivity. Animals.

[80] Muralidharan M.R., Ramesh V. (2005). Histological and biochemical studies of the skin of cattle and buffalo. Indian J. Anim. Res..

[81] Debbarma D., Uppal V., Bansal N., Gupta A. (2018). Histomorphometrical study on regional variation in distribution of sweat glands in buffalo skin. Dermatol. Res. Pract..

[82] Lendez P.A., Martínez Cuesta L., Nieto Farías M.V., Vater A.A., Ghezzi M.D., Mota-Rojas D., Dolcini G.L., Ceriani M.C. (2023). Effect of heat stress on TNF-α, TNFRI and TNFRII expression in BLV infected dairy cattle. J. Therm. Biol..

[83] Shafies M.M., El-Khair M.M.A. (1970). Activity of the sebaceous glands of bovines in hot climates. United Arab Repub. J. Anim. Prod..

[84] Mota-Rojas D., Habeeb A., Ghezzi M.D., Kanth P., Napolitano F., Lendez P.A., Cuibus A., Ceriani M.C., Sarubbi J., Braghieri A., Napolitano F., Mota-Rojas D., Guerrero-Legarreta I., Orihuela A. (2020). Termorregulación del búfalo de agua: Mecanismos neurobiológicos, cambios microcirculatorios y aplicaciones prácticas de la termografía infrarroja. El Búfalo de Agua en Latinoamérica, Hallazgos Recientes.

[85] Flinn T., Kleemann D.O., Swinbourne A.M., Kelly J.M., Weaver A.C., Walker S.K., Gatford K.L., Kind K.L., van Wettere W.H.E.J. (2020). Neonatal lamb mortality: Major risk factors and the potential ameliorative role of melatonin. J. Anim. Sci. Biotechnol..

[86] Bienboire-Frosini C., Muns R., Marcet-Rius M., Gazzano A., Villanueva-García D., Martínez-Burnes J., Domínguez-Oliva A., Lezama-García K., Casas-Alvarado A., Mota-Rojas D. (2023). Vitality in Newborn Farm Animals: Adverse Factors, Physiological Responses, Pharmacological Therapies, and Physical Methods to Increase Neonate Vigor. Animals.

[87] Lezama-García K., Martínez-Burnes J., Baquerio-Espinosa U., Olmos-Hernández A., Hernández-Avalos I., Domínguez-Oliva A., Mota-Rojas D. (2023). Assessment of vitality, blood profile, and degree of meconium staining on the skin in neonate dogs according to its birth weight. Vet. Sci..

[88] Mülling M. (1976). Asphyxie des neugeborenen Kalbes. Prakt. Tierarzt Coll..

[89] Szenci O. (2023). Importance of monitoring fetal and neonatal vitality in bovine practices. Animals.

[90] Mee J.F. (2008). Newborn dairy calf management. Vet. Clin. North Am. Food Anim. Pract..

[91] Nel C.L., Cloete S.W.P., Kruger A.C.M., Dzama K. (2021). Long term genetic selection for reproductive success affects neonatal lamb vitality across cold stress conditions. J. Therm. Biol..

[92] Probo M., Veronesi M.C. (2022). Clinical scoring systems in the newborn calf: An overview. Animals.

[93] Afzal M.M.H., Anjum A. (1983). Calf mortality: Season pattern, age distribution and causes of calf mortality. Pak. Vet. J..

[94] Vannucchi C.I., Rodrigues J.A., Silva L.C.G., Lúcio C.F., Veiga G.A.L. (2015). Effect of dystocia and treatment with oxytocin on neonatal calf vitality and acid-base, electrolyte and haematological status. Vet. J..

[95] Vannucchi C.I.C., Rodrigues J.J.A., Silva L.L.C.G., Lúcio C.C.F., Veiga G.A.L.G. (2012). A clinical and hemogasometric survey of neonatal lambs. Small Rumin. Res..

[96] Murray C.F., Leslie K.E. (2013). Newborn calf vitality: Risk factors, characteristics, assessment, resulting outcomes and strategies for improvement. Vet. J..

[97] Mota-Rojas D., Villanueva-García D., Hernández-Ávalos I., Casas-Alvarado A., Domínguez-Oliva A., Lezama-García K., Miranda-Cortés A., Martínez-Burnes J. (2023). Cardiorespiratory and Neuroprotective Effects of Caffeine in Neonate Animal Models. Animals.

[98] Lanzoni L., Chincarini M., Giammarco M., Fusaro I., Gloria A., Contri A., Ferri N., Vignola G. (2021). Maternal and neonatal behaviour in italian mediterranean buffaloes. Animals.

[99] Singh A., Prachakar S., Brar P., Uppal S., Singh P., Gandotra V. (2011). Blood biochemical profiles and physical-activity parameters in neonatal buffalo calves under normal and forced calving. Indian J. Anim. Sci..

[100] Diesch T., Mellor D., Stafford K., Ward R. (2004). The physiological and physical status of single calves at birth in a dairy herd in New Zealand. N. Z. Vet. J..

[101] Haughey K.G. (1973). Cold injury in newborn lambs. Aust. Vet. J..

[102] Graña-Baumgartner A., Dukkipati V.S.R., Kenyon P.R., Blair H.T., López-Villalobos N., Gedye K., Biggs P.J. (2022). RNAseq Analysis of brown adipose tissue and thyroid of newborn lambs subjected to short-term cold exposure reveals signs of early whitening of adipose tissue. Metabolites.

[103] Sreedhar S., Ranganadham M., Mohan E.M. (2010). Calf mortality in indigenous buffaloes. Indian Vet. J..

[104] Torell R., Kvasnicke B., Bruce B. Care of Hypothermic (Cold Stressed) Newborn Beef Cows. https://www.thecattlesite.com/articles/1317/caring-for-hypothermic-cold-stressed-newborn-beef-calves.

[105] Yáñez-Pizaña A., de la Cruz-Cruz L.A., Tarazona-Morales A., Roldan-Santiago P., Ballesteros-Rodea G., Pineda-Reyes R., Orozco-Gregorio H. (2020). Physiological and behavioral changes of water buffalo in hot and cold systems: Review. J. Buffalo Sci..

[106] Da Silva J.A.R., de Andrade Pantoja M.H., da Silva W.C., de Almeida J.C.F., de Paula PachechoNoronha R., Barbosa A.V.C., de Brito Lourenço Júnior J. (2022). Thermoregulatory reactions of female buffaloes raised in the sun and in the shade, in the climatic conditions of the rainy season of the Island of Marajó, Pará, Brazil. Front. Vet. Sci..

[107] Zhao W., Choi C., Li D., Yan G., Li H., Shi Z. (2021). Effects of airspeed on the respiratory rate, rectal temperature, and immunity parameters of dairy calves housed individually in an axial-fan-ventilated barn. Animals.

[108] Roland L., Drillich M., Klein-Jöbstl D., Iwersen M. (2016). Invited review: Influence of climatic conditions on the development, performance, and health of calves. J. Dairy Sci..

[109] Hill T.M., Bateman H.G., Suarez-Mena F.X., Dennis T.S., Schlotterbeck R.L. (2016). Short communication: Changes in body temperature of calves up to 2 months of age as affected by time of day, age, and ambient temperature. J. Dairy Sci..

[110] Merlet C., Leandri J., Rey P., Tchobroutsky C. (1967). Effect of localized cooling on starting respiration in lambs at birth. J. Physiol..

[111] Harned H.S., Herrington R.T., Ferreiro J.I. (1970). The effects of immersion and temperature on respiration in newborn lambs. Pediatrics.

[112] Farrag B. (2022). Effect of seasonal variations during dry and wet seasons on reproductive performance and biological and economic criteria of hair sheep under Halaieb rangeland conditions. Arch. Anim. Breed..

[113] Hulbert L.E., Moisá S.J. (2016). Stress, immunity, and the management of calves. J. Dairy Sci..

[114] Buijs T.J., McNaughton P.A. (2020). The role of cold-sensitive ion channels in peripheral thermosensation. Front. Cell. Neurosci..

[115] McKemy D.D. (2005). How Cold is It? TRPM8 and TRPA1 in the molecular logic of cold sensation. Mol. Pain.

[116] Zhao Z.-D., Yang W.Z., Gao C., Fu X., Zhang W., Zhou Q., Chen W., Ni X., Lin J.-K., Yang J. (2017). A hypothalamic circuit that controls body temperature. Proc. Natl. Acad. Sci. USA.

[117] Rothhaas R., Chung S. (2021). Role of the preoptic area in sleep and thermoregulation. Front. Neurosci..

[118] Guseynov N.A., Hammouri M.H., Muraev A.A., Ivanov S.Y., Lukianova E.A., Klimenko A.S., Noeerazlighi M.A. (2022). Local hardware hypothermia influence on the physiological processes. Rudn. J. Med..

[119] Polli V.A., Vaz R.Z., Carvalho S., Costa P.T., de Oliveira Mello R., Restle J., Nigeliskii A.F., Silveira I.D.B., Pissinin D. (2019). Thermal comfort and performance of feedlot lambs finished in two climatic conditions. Small Rumin. Res..

[120] Van Someren E.J.W. (2011). Age-related changes in thermoreception and thermoregulation. Handbook of the Biology of Aging.

[121] Vilela R.A., de Brito Lourenço Junior J., Jacintho M.A.C., Barbosa A.V.C., Pantoja M.H.d.A., Oliveira C.M.C., Garcia A.R. (2022). Dynamics of thermolysis and skin microstructure in water buffaloes reared in humid tropical climate—A microscopic and thermographic study. Front. Vet. Sci..

[122] Pressicce G.A. (2017). The Buffalo (Bubalus bubalis): Production and Research.

[123] Nazari S., Kourosh-Arami M., Komaki A., Hajizadeh S. (2020). Relative contribution of central and peripheral factors in superficial blood flow regulation following cold exposure. Physiol. Pharmacol..

[124] Slee J. (1968). Body temperature and vasomotor responses in Scottish Blackface and Tasmanian Merino sheep subjected to slow cooling. Anim. Sci..

[125] Turnpenny J., Wathes C., Clark J., McArthur A. (2000). Thermal balance of livestock. Agric. For. Meteorol..

[126] Bianco A.C., Silva J.E. (1987). Intracellular conversion of thyroxine to triiodothyronine is required for the optimal thermogenic function of brown adipose tissue. J. Clin. Invest..

[127] Alexander G. (1981). Development of brown fat in the sheep fetus: Preparation for neonatal thermogenesis. Proc. Aust. Physiol. Pharmacol. Soc..

[128] Gunn T.R., Gluckman P.D. (1995). Perinatal thermogenesis. Early Hum. Dev..

[129] Cannon B., Romert L., Sundin U., Barnard T. (1977). Morphology and biochemical properties of perirenal adipose tissue from lamb (Ovis aries). A comparison with brown adipose tissue. Comp. Biochem. Physiol. Part B Comp. Biochem..

[130] Clarke L., Symonds M.E. (1998). Thermoregulation in newborn lambs: Influence of feeding and ambient temperature on brown adipose tissue. Exp. Physiol..

[131] Nowack J., Vetter S.G., Stalder G., Painer J., Kral M., Smith S., Le M.H., Jurcevic P., Bieber C., Arnold W. (2019). Muscle nonshivering thermogenesis in a feral mammal. Sci. Rep..

[132] Louveau I., Perruchot M.-H., Bonnet M., Gondret F. (2016). Invited review: Pre- and postnatal adipose tissue development in farm animals: From stem cells to adipocyte physiology. Animal.

[133] Soppela P., Sormunen R., Saarela S., Huttunen P., Nieminen M. (1992). Localization, cellular morphology and respiratory capacity of “brown” adipose tissue in newborn reindeer. Comp. Biochem. Physiol. Part A Physiol..

[134] Carstens G.E. (1994). Cold thermoregulation in the newborn calf. Vet. Clin. North Am. Food Anim. Pract..

[135] Tansey E.A., Johnson C.D. (2015). Recent advances in thermoregulation. Adv. Physiol. Educ..

[136] Legendre L.J., Davesne D. (2020). The evolution of mechanisms involved in vertebrate endothermy. Philos. Trans. R. Soc. B Biol. Sci..

[137] Hohtola E., Barnes M., Carey H.V. (2004). Shivering Thermogenesis in Birds and Mammals. Life in the Cold: Evolution, Mechanisms, Adaptation, and Application.

[138] Haman F. (2006). Shivering in the cold: From mechanisms of fuel selection to survival. J. Appl. Physiol..

[139] Rowland L.A., Bal N.C., Periasamy M. (2015). The role of skeletal-muscle-based thermogenic mechanisms in vertebrate endothermy. Biol. Rev..

[140] Mila H., Feugier A., Grellet A., Anne J., Gonnier M., Martin M., Rossig L., Chastant-Maillard S. (2015). Immunoglobulin G concentration in canine colostrum: Evaluation and variability. J. Reprod. Immunol..

[141] Kienzle E., Zentek J., Meyer H. (1998). Body composition of puppies and young dogs. J. Nutr..

[142] Berthon D., Herpin P., Bertin R., De Marco F., le Dividich J. (1996). Metabolic changes associated with sustained 48-Hr shivering thermogenesis in the newborn pig. Comp. Biochem. Physiol. Part B Biochem. Mol. Biol..

[143] Curtis S. (1970). Environmental—Thermoregulatory interactions and neonatal piglet survival. J. Anim. Sci..

[144] Herpin P., Vincent A., Damon M. (2004). Effect of breed and body weight on thermoregulatory abilities of European (Piétrain×(Landrace×Large White)) and Chinese (Meishan) piglets at birth. Livest. Prod. Sci..

[145] Mota-Rojas D., Gregorio O., Villanueva D., Bonilla H., Suárez X., Hernandez R., Roldan P., Trujillo M. (2011). Foetal and neonatal energy metabolism in pigs and humans: A review. Vet. Med..

[146] Alexander G., Williams D. (1968). Shivering and non-shivering thermogenesis during summit metabolism in young lambs. J. Physiol..

[147] Smith S., Carstens G., Burrin D., Mersmann H. (2005). Ontogeny and metabolism of brown adipose tissue in livestock species. Biology of Growing Animals.

[148] Lourenço M.L.G., Machado L.H.A. (2013). Particularidades do período de transição fetal-neonatal em neonatos caninos. Rev. Bras. Reprod. Anim.

[149] Rödel H.G., Bautista A., García-Torres E., Martínez-Gómez M., Hudson R. (2008). Why do heavy littermates grow better than lighter ones? A study in wild and domestic European rabbits. Physiol. Behav..

[150] García-Torres E., Hudson R., Castelán F., Martínez-Gómez M., Bautista A. (2015). Differential metabolism of brown adipose tissue in newborn rabbits in relation to position in the litter huddle. J. Therm. Biol..

[151] Carlton P., Marks R. (1958). Cold exposure and heat reinforced operant behavior. Science.

[152] Terrien J., Perret M., Aujard F. (2011). Behavioral thermoregulation in mammals: A review. Front. Biosci..

[153] Ferner K., Schultz J.A., Zeller U. (2017). Comparative anatomy of neonates of the three major mammalian groups (monotremes, marsupials, placentals) and implications for the ancestral mammalian neonate morphotype. J. Anat..

[154] Hrupka B.J., Leibbrandt V.D., Crenshaw T.D., Benevenga N.J., Hrupka B.J., Leibbrandt V.D., Crenshaw T.D., Benevenga N.J. (2000). Effect of sensory stimuli on huddling behavior of pigs. J. Anim. Sci..

[155] Lezama-García K., Mariti C., Mota-Rojas D., Martínez-Burnes J., Barrios-García H., Gazzano A. (2019). Maternal behaviour in domestic dogs. Int. J. Vet. Sci. Med..

[156] Dwyer C.M., Conington J., Corbiere F., Holmøy I.H., Muri K., Nowak R., Rooke J., Vipond J., Gautier J.-M. (2016). Invited review: Improving neonatal survival in small ruminants: Science into practice. Animal.

[157] Harri M., Mononen J., Haapanen K., Korhonen H. (1991). Postnatal changes in hypothermic response in farmborn blue foxes and raccoon dogs. J. Therm. Biol..

[158] Coroian A., Erler S., Matea C.T., Mireșan V., Răducu C., Bele C., Coroian C.O. (2013). Seasonal changes of buffalo colostrum: Physicochemical parameters, fatty acids andcholesterol variation. Chem. Cent. J..

[159] Scheuer B.H., Zbinden Y., Schneiter P., Tappy L., Blum J.W., Hammon H.M. (2006). Effects of colostrum feeding and glucocorticoid administration on insulin-dependent glucose metabolism in neonatal calves. Domest. Anim. Endocrinol..

[160] Souza D.C., Silva D.G., Rocha T.G., Monteiro B.M., Pereira G.T., Fiori L.C., Viana R.B., Fagliari J.J. (2019). Serum biochemical profile of neonatal buffalo calves. Arq. Bras. Med. Vet. Zootec..

[161] Quigley J.D., Drewry J.J. (1998). Nutrient and immunity transfer from cow to calf pre- and postcalving. J. Dairy Sci..

[162] Ojha B.K., Dutta N., Pattanaik A.K., Singh S.K., Narang A. (2015). Effect of pre-partum strategic supplementation of concentrates on colostrum quality and performance of buffalo calves. Anim. Nutr. Feed Technol..

[163] Dang A.K., Kapila S., Purohit M., Singh C. (2009). Changes in colostrum of Murrah buffaloes after calving. Trop. Anim. Health Prod..

[164] Farmer C., Quesnel H. (2009). Nutritional, hormonal, and environmental effects on colostrum in sows. J. Anim. Sci..

[165] Guy M.A., McFadden T.B., Cockrell D.C., Besser T.E. (1994). Regulation of colostrum formation in beef and dairy cows. J. Dairy Sci..

[166] Castro N., Capote J., Bruckmaier R.M., Argüello A. (2011). Management effects on colostrogenesis in small ruminants: A review. J. Appl. Anim. Res..

[167] Erdem H., Okuyucu I.C. (2020). Non-genetic factors affecting some colostrum quality traits in Holstein cattle. Pak. J. Zool..

[168] Mellor D., Murray L. (1986). Making the most of colostrum at lambing. Vet. Rec..

[169] McGee M., Drennan M.J., Caffrey P.J. (2006). Effect of age and nutrient restriction pre partum on beef suckler cow serum immunoglobulin concentrations, colostrum yield, composition and immunoglobulin concentration and immune status of their progeny. Irish J. Agric. Food Res..

[170] Abdel-Hamid M., Yang P., Mostafa I., Osman A., Romeih E., Yang Y., Huang Z., Awad A.A., Li L. (2022). Changes in whey proteome between Mediterranean and Murrah buffalo colostrum and mature milk reflect their pharmaceutical and medicinal value. Molecules.

[171] Ashmawy N. (2015). Chemical composition, hormonal levels and immunoglobin G concentration in colostrums, milk and blood plasma of Egyptian buffaloes following calving. Int. J. Adv. Res..

[172] Qureshi T.M., Yaseen M., Nadeem M., Murtaza M.A., Munir M. (2020). Physico–chemical composition and antioxidant potential of buffalo colostrum, transition milk, and mature milk. J. Food Process. Preserv..

[173] Abd El-Fattah A.M., Abd Rabo F.H., EL-Dieb S.M., El-Kashef H.A. (2012). Changes in composition of colostrum of Egyptian buffaloes and Holstein cows. BMC Vet. Res..

[174] An Z., Luo G., Gao S., Zhang X., Chen C., Yao Z., Zhao J., Lv H., Niu K., Nie P. (2023). Evaluation of parity effect on characteristics and minerals in buffalo (*Bubalus Bubalis*) colostrum and mature milk. Foods.

[175] Barmaiya S., Dixit A., Mishra A., Jain A.K., Gupta A., Paul A., Quadri M.A., Madan A.K., Sharma I.J. (2009). Quantitation of serum immunoglobulins of neonatal buffalo calves and cow calves through elisa and page: Status of immune-competence. Buffalo Bull..

[176] Erdem H., Okuyucu I.C., Demirci H. (2022). Components and specific gravity of colostrum from Anatolian buffalo cows and effects on growth of buffalo calves. S. Afr. J. Anim. Sci..

[177] Silva F.L.M., Miqueo E., da Silva M.D., Torrezan T.M., Rocha N.B., Salles M.S.V., Bittar C.M.M. (2021). Thermoregulatory responses and performance of dairy calves fed different amounts of colostrum. Animals.

[178] Wang F.-K., Shih J.-Y., Juan P.-H., Su Y.-C., Wang Y.-C. (2021). Non-invasive cattle body temperature measurement using infrared thermography and auxiliary sensors. Sensors.

[179] Tattersall G.J., Cadena V. (2010). Insights into animal temperature adaptations revealed through thermal imaging. Imaging Sci. J..

[180] Giro A., de Campos Bernardi A.C., Barioni Junior W., Lemes A.P., Botta D., Romanello N., Barreto A.d.N., Garcia A.R. (2019). Application of microchip and infrared thermography for monitoring body temperature of beef cattle kept on pasture. J. Therm. Biol..

[181] Tattersall G.J. (2016). Infrared thermography: A non-invasive window into thermal physiology. Comp. Biochem. Physiol. Part A Mol. Integr. Physiol..

[182] Casas-Alvarado A., Martínez-Burnes J., Mora-Medina P., Hernández-Avalos I., Domínguez-Oliva A., Lezama-García K., Gómez-Prado J., Mota-Rojas D. (2022). Thermal and circulatory changes in diverse body regions in dogs and cats evaluated by infrared thermography. Animals.

[183] Dela Ricci G., Silva-Miranda K.O., Titto C.G. (2019). Infrared thermography as a non-invasive method for the evaluation of heat stress in pigs kept in pens free of cages in the maternity. Comput. Electron. Agric..

[184] Stewart M., Wilson M.T., Schaefer A.L., Huddart F., Sutherland M.A. (2017). The use of infrared thermography and accelerometers for remote monitoring of dairy cow health and welfare. J. Dairy Sci..

[185] Lowe G., Sutherland M., Waas J., Schaefer A., Cox N., Stewart M. (2019). Infrared thermography—A non-invasive method of measuring respiration rate in calves. Animals.

[186] Shu H., Li Y., Fang T., Xing M., Sun F., Chen X., Bindelle J., Wang W., Guo L. (2022). Evaluation of the best region for measuring eye temperature in dairy cows exposed to heat stress. Front. Vet. Sci..

[187] Sutherland M.A., Worth G.M., Dowling S.K., Lowe G.L., Cave V.M., Stewart M. (2020). Evaluation of infrared thermography as a non-invasive method of measuring the autonomic nervous response in sheep. PLoS ONE.

[188] Labeur L., Villiers G., Small A.H., Hinch G.N., Schmoelzl S. (2017). Infrared thermal imaging as a method to evaluate heat loss in newborn lambs. Res. Vet. Sci..

[189] Bertoni A., Mota-Rojas D., Álvarez-Macias A., Mora-Medina P., Guerrero-Legarreta I., Morales-Canela A., Gómez-Prado J., José-Pérez N., Martínez-Burnes J. (2020). Scientific findings related to changes in vascular microcirculation using infrared thermography in the river buffalo. J. Anim. Behav. Biometeorol..

[190] Manani M., Jegatheesan P., DeSandre G., Song D., Showalter L., Govindaswami B. (2013). Elimination of admission hypothermia in preterm very low-birth-weight infants by standardization of delivery room management. Perm. J..

[191] Islas P., Mota-Rojas D., Martínez-Burnes J., Mora-Medina P., González-Lozano M., Santiago R., Greenwell-Beare V., González-Hernández M., Vega-Manríquez X., Gregorio O. (2018). Physiological and metabolic responses in newborn piglets associated with the birth order. Anim. Reprod. Sci..

[192] Gloria A., Chincarini M., Vignola G., Ferri N., Contri A. (2020). Venous blood gas parameters in healthy Mediterranean buffalo calves in the first 72 hours of life. Theriogenology.

[193] Rodríguez-González D., Guerrero-Legarreta I., Cruz Monterrosa R.G., Napolitano F., Gonçalves-Titto C., El-Aziz A.H.A., Hernández-Ávalos I., Casas-Alvarado A., Oliva- Domínguez A., Mota-Rojas D. (2023). Assessment of thermal changes in water buffalo mobilized from the paddock and transported by short journeys. Front. Vet. Sci..

